# Structure, evolution, and roles of MYB transcription factors proteins in secondary metabolite biosynthetic pathways and abiotic stresses responses in plants: a comprehensive review

**DOI:** 10.3389/fpls.2025.1626844

**Published:** 2025-07-31

**Authors:** Ziming Ma, Lanjuan Hu, Yu Zhong

**Affiliations:** ^1^ Jilin Provincial Engineering Laboratory of Plant Genetic Improvement, College of Plant Science, Jilin University, Changchun, China; ^2^ Root Biology and Symbiosis, Max Planck Institute of Molecular Plant Physiology, Potsdam, Germany

**Keywords:** MYB transcription factor, plant growth and development, abiotic stress, phytohormone signaling, secondary metabolic synthetic pathways

## Abstract

Unlike mobile organisms, plants are sessile and thus more vulnerable to environmental stressors. Among these, abiotic stress represents a major constraint that profoundly affects plant growth and development. To cope with these challenges, plants have evolved sophisticated adaptive mechanisms to enhance their stress resilience. Transcription factors (TFs) play a pivotal role in these adaptive processes, as they are activated by diverse stress signals and subsequently modulate the expression of stress-responsive genes, thereby improving plant survival under adverse conditions. The MYB TF family, one of the largest TF families in plants, participates in regulating various biological processes, including growth and development, phytohormone signaling, secondary metabolism and abiotic stress responses. Numerous studies have demonstrated that MYB TFs, upon activation by environmental stimuli, can bind to cis-acting elements in the promoters of downstream stress-responsive genes or interact with other proteins to fine-tune their expression, ultimately enhancing plant tolerance to abiotic stress. Additionally, MYB TFs are integral components of phytohormone signaling pathways involved in stress adaptation. Although extensive research has been conducted on plant stress responses, the interplay between MYB TFs and phytohormones in mediating abiotic stress tolerance remains underexplored. In this review, we examine the structural features, classification, and functional mechanisms of MYB transcription factors. Furthermore, we summarize current knowledge on the roles of MYB TFs (both hormone-dependent and hormone-independent) in plant responses to various abiotic stresses, including drought, salinity, extreme temperatures, nutrient deficiencies, and heavy metal toxicity. We also discuss their regulatory roles in the biosynthesis of secondary metabolites, such as glucosinolates, flavonoids, terpenoids, lignans, and astragalosides. In conclusion, this review consolidates existing findings and provides a foundation for uncovering novel functions and regulatory mechanisms of the MYB TF family. Future research should prioritize MYB TFs as central regulators of abiotic stress-responsive gene networks, with the potential to improve crop stress tolerance and yield, thereby addressing global food security challenges.

## 1 Introduction

**Figure 1:**
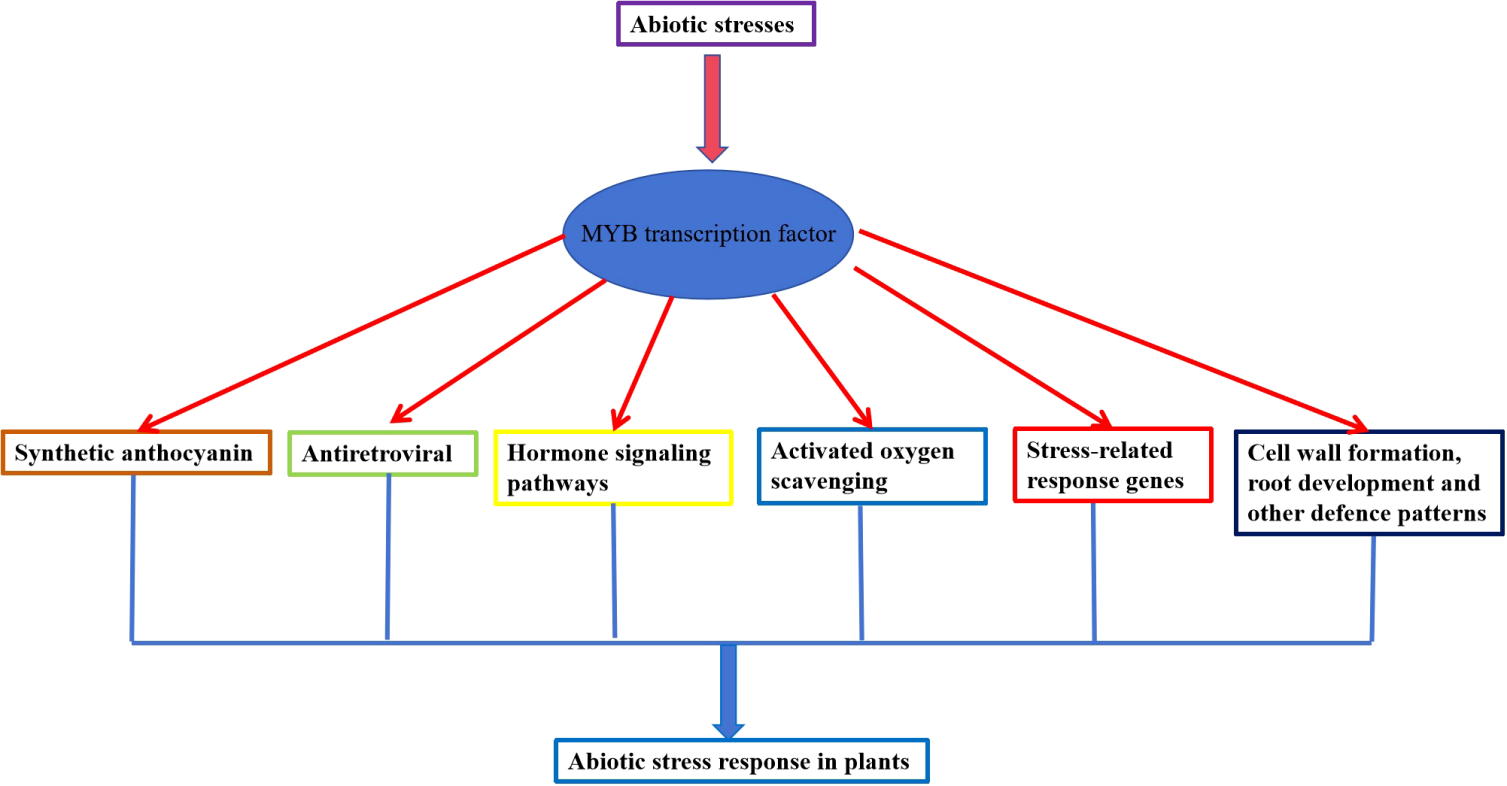
MYB transcription factors involved in the regulatory mode in plants abiotic stress response.

Transcription factors (IFs) play important roles in human and animals, especially in higher plants for the regulation of plant growth and development, adversity stress, and damage defense (Liu et al., 2010; Bayliak et al., 2020; Shan et al., 2014). Structurally, TFs typically contain DNA-binding domains, transcriptional regulatory domains, oligomerization sites, and nuclear localization signals. They modulate gene expression by interacting with other TFs or binding to promoter sequences of downstream genes, thereby enhancing or suppressing transcription. This regulatory mechanism is essential for improving plant tolerance to abiotic stresses and coordinating physiological and biochemical processes throughout the plant life cycle (Ma et al., 2020, 2022; Ma and Hu, 2023, 2024a; Ma et al., 2024b). MYB (v-myb avian myeloblastosis viral oncogene homolog) transcription factors are one of the largest transcription factor families in plants. It is widely involved in various important biological processes of plants, such as plant growth and development, cell formation and differentiation, primary and secondary metabolism, and response to biotic and abiotic stresses (Liu et al., 2015; Pratyusha and Sarada, 2022; Ambawat et al., 2013; Wang et al., 2021a) (**Figure 1**). In 1982, a MYB TF gene (*v-MYB*) was found in avian myeloblastosis virus (AMV) (Klempnauer et al., 1982). In 1987, Pazares et al. isolated and identified the first MYB transcription factor Clorless1 from *Zea mays* in plants, which can participate in the biosynthesis of anthocyanins in maize (Pazares et al., 1987). Then Weston isolated and identified three *v-MYB* related genes in vertebrates, namely *c*-*MYB*, *A*-*MYB* and *B*-*MYB*, which were confirmed to regulate cell proliferation, tissue differentiation and cell death (Weston, 1998). So far, the number of MYB TF genes found in plants are much higher than fungi and animals (Riechmann et al., 2000). With the publication of *Arabidopsis* genome sequence, the classification of MYB genes in plants has been comprehensively elaborated for the first time (Stracke et al., 2001). Recent research highlights the pivotal role of MYB TFs in plant stress responses. Upon activation by environmental signals, they bind to cis-acting elements (e.g., MYBCORE, AC-box, P-box, H-box, G-box) in target gene promoters, either independently or in complexes with other proteins, to modulate stress-responsive gene expression (Chen et al., 2022c; Zhang et al., 2025).

According to the number of DNA binding domains and incomplete repeats, MYB proteins are divided into four types: R1, R2R3, R3 and R4 (Dubos et al., 2010). Four types of MYB TF jointly regulate multiple functions, such as secondary metabolism, cell cycle control, development process and stress response. R1-MYB protein: CIRCADIAN CLOCK ASSOCIATED1 (CCA1) and LATE-elongated HYPOCOTYL (LHY) MYB protein that function in or close to the central oscillator in *Arabidopsis* (Lu et al., 2009). R2R3-MYB protein: the specific functions of R2R3-MYB protein include signal transduction in response to abiotic stresses such as cold, drought, light, nutrient deficiency, and ultraviolet radiation. The *Arabidopsis* R2R3-MYB gene mutant *Atmyb4* exhibits enhanced levels of sinapine in its leaves. The mutant strain is more tolerant to UV-B radiation than wild type. UV-B light can downregulate the expression of *AtMYB4* in *Arabidopsis* (Jin et al., 2000; Miyake et al., 2003; Chen et al., 2005). R3-MYB protein: Most R3-MYB proteins are involved in salt stress and drought stress in plants. Jin et al. found two R3 MYB TF genes *OsTCL1* and *OsTCL2* in *Oryza sativa* L. The mutation alleles of *tcl1* and *tcl2* delayed seed germination, especially under stress condition. The germination rate of *tcl1-tcl2* double mutant decreased more significantly in *Oryza sativa* L (Yi et al., 2023). R4-MYB protein: Thiedig et al. found AtSNAPc4 belongs to R4-MYB protein, which is involved in the induction of small nuclear RNA gene transcription as an intranuclear component of atsnapc complex. The *snapc4* mutant showed exhibited defects in the gametophytic functions, pollen viability, and the transmission efficiency, possibly due to misexpression of small nuclear RNA genes (Thiedig et al., 2021). In summary, the unique characteristics and pivotal functions of MYB transcription factors have attracted significant scientific interest, prompting extensive research into their roles across diverse plant species.

Over the past five years, several reviews on MYB transcription factors have been published. However, most existing reviews primarily focus on their discovery, structural features, classification, functional diversity, and regulatory mechanisms under specific stress conditions. These studies provide limited insights into the comprehensive regulatory networks of MYB transcription factors, leaving critical gaps in our understanding. Wang et al (Wang et al., 2024b). reviewed the structure and classification of MYB transcription factors, biotic and abiotic stress tolerance and their roles in cotton secondary metabolism. Wu et al (Wu et al., 2024). reviewed the structure, classification and biological functions of MYB TFs, with special focus on their roles and mechanisms in response to biotic and abiotic stresses, plant morphogenesis and secondary metabolite biosynthesis. Bhatt et al (Bhatt et al., 2025). summarized the structural and functional differences between activator and repressor MYB proteins and their roles in plant growth and development, stress response and secondary metabolite production. Therefore, there is little literature summarizing the influence of MYB transcription factors on plant stress tolerance signaling pathways through hormonal pathways.

Therefore, it is necessary to conduct a more comprehensive review of plant MYB transcription factors, which will provide a comprehensive perspective for the in-depth study of MYB transcription factors. To fill this knowledge gap, This review systematically addresses several key aspects: (1) the structure, classification, and evolution of MYB transcription factors; (2) their involvement in plant hormone signaling and abiotic stress responses; (3) their mechanisms of action in enhancing plant stress resistance. (4) the role of MYB transcription factors in plant secondary metabolism, summarizing prior research methodologies and highlighting recent advancements. (5) current research challenges and propose future directions for investigation. By integrating these insights, this review serves as a valuable resource for elucidating the functions of MYB transcription factors in hormone regulation, abiotic stress adaptation, and secondary metabolism. The findings presented herein not only contribute to stress-resistant crop breeding but also provide essential genetic resources and theoretical foundations for improving abiotic stress tolerance in agricultural applications.

### 1.1 The structure and classification of the plant MYB transcription factors

**Figure 2:**
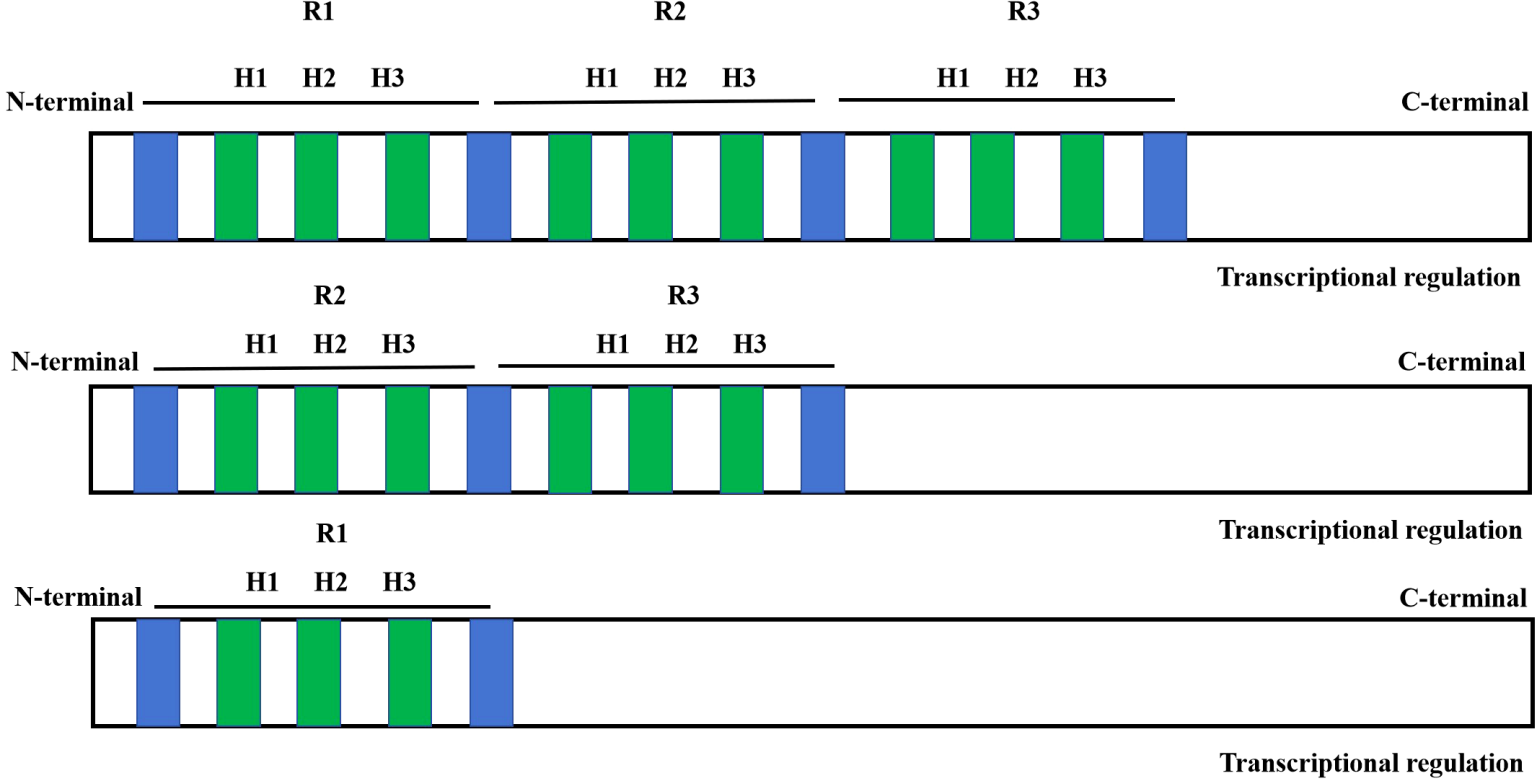
Domain structures of MYB transcription factors. R1, R2 and R3 represent repeated myb binding domains. The blue part indicates the conserved DNA junction domain in the myb protein structure. The green part of MYB domain represents three α - helices. The transcriptional activation domain of MYB protein is located at the C-terminal.

The MYB transcription factor family is a group of highly conserved DNA-binding domains known as MYB transcription factor structural domains. Each MYB transcription factor contains one to four repetitive MYB domains. Each repeat has approximately 52 amino acid residues, which are inserted into the main groove of double-stranded DNA in a helix-rotate-helix conformation. Each MYB repeat contains three α-helix connected by a corner between the second and third helices to form a stable helix-turn-helix (H-T-H). In the MYB domain sequence, there is a tryptophan residue (W) between every 18–19 amino acid residues, whose main function is to form a hydrophobic core in the H-T-H three-dimensional structure. The HTH three-dimensional structure consists of three regularly spaced hydrophobic amino acids, usually Trp, which are sometimes replaced by Phe or Leu, and which form a hydrophobic core, which is critical for the maintenance of the spatial conformation of MYBs. The third helix is considered to be the ‘recognition helix’ and is responsible for recognizing the DNA binding site and structurally binding to the target DNA in the major groove (**Figure 2**) (Yang et al., 2022a).

**Figure 3:**
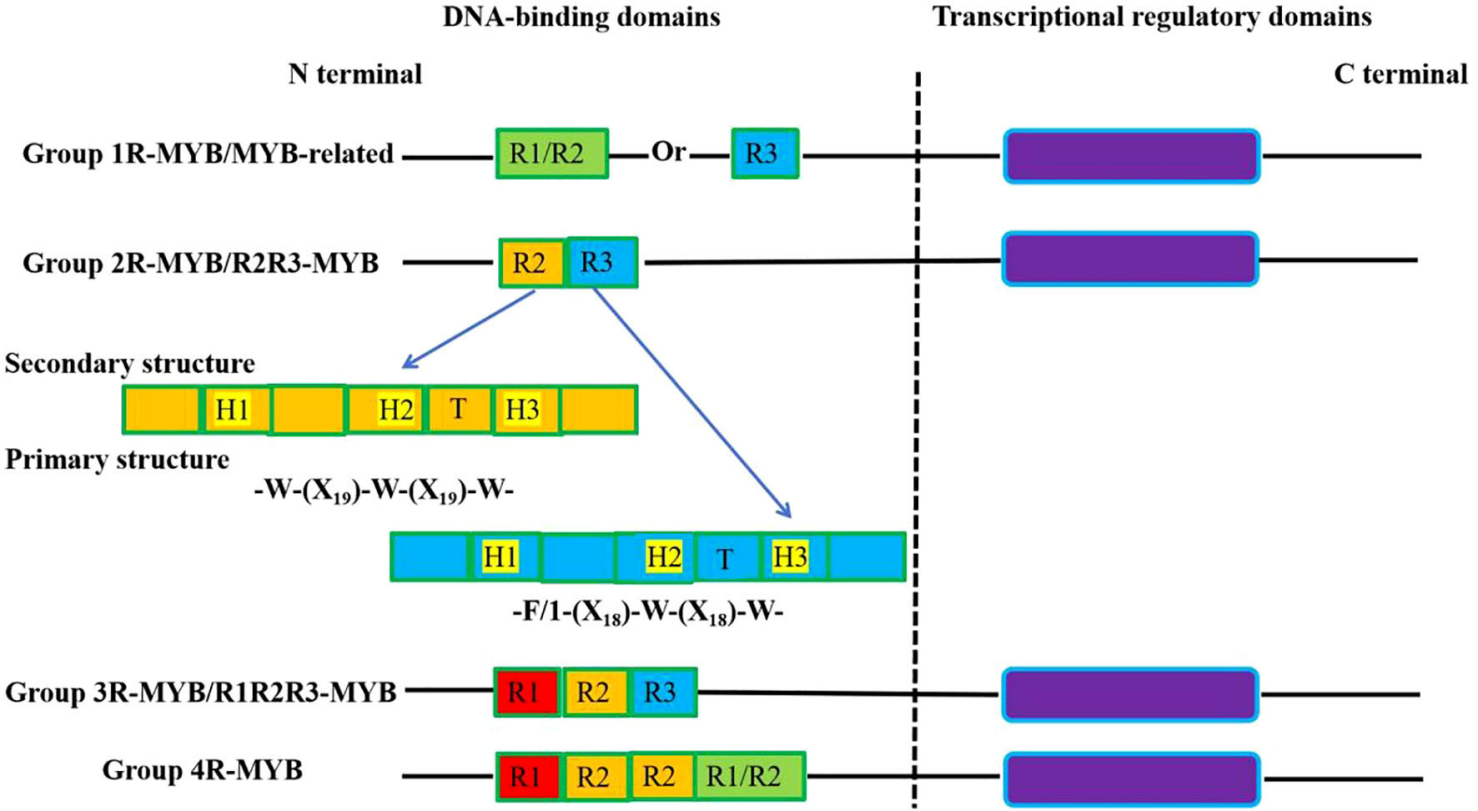
Domains structure of MYB family transcription factors in plants. MYB TFs are classified as 1R-MYB, R2R3-MYB (majority), R3-MYB, R4-MYB and MYB-like (MYB-related). R1, R2, and R3 are the MYB domain, where H1-H3 indicate the α-helix and T indicate the β-turn. W: Trp; F: Phe; I: Ile; X: amino acid.

The presence of three regularly spaced tryptophan in each MYB repeat sequence forms a hydrophobic cluster that is associated with specific recognition of the DNA sequence (Li et al., 2022b). Based on the similarity to the three repeat sequences R1, R2, or R3 in animal c-myb and the number of R repeat sequences, the plant MYB TFs family has been classified into four categories: MYB TFs are classified as 1R-MYB, R2R3-MYB (majority), R3-MYB, R4-MYB and MYB-like (MYB-related) (**Figure 3**) (Dubos et al., 2010).

In 1987, scientists cloned the first plant MYB-like transcription factor gene *COLORED1* from maize. Its encoded protein ZmMYBC1 and found that ZmMYBC1 is mainly involved in anthocyanin synthesis, and nowadays it is one of the most abundant classes of MYB TFs in plants (Pazares et al., 1987). Nowadays, researchers have found from *Arabidopsis thaliana* L., *Capsicum annuum* L., *Spinacia oleracea* L., *Oryza sativa* L., and other species (**Table 1**). A large number of MYB genes have been identified in plants. So far, a cumulative total of 198 MYB genes have been identified in the *Arabidopsis thaliana* L. genome and a cumulative total of 239 MYB genes have been identified in the *Oryza sativa* genome. From the table, we can find that the number of MYB family members in different species showed some differences, but the number of MYB genes encoding R2R3-MYB was higher than that of other MYB genes in most species. Thus, R2R3-MYB is the most abundant subclass of the MYB family in most plants, and it exists in many monocotyledonous and dicotyledonous plants. The MYB transcription factors are widely involved in plant growth and development, cell differentiation, metabolic pathway regulation, and abiotic stress response (Muthuramalingam et al., 2022; Liu et al., 2024b; Prusty et al., 2024; Wu et al., 2022).

**Table 1:** The MYBs genes total numbers in different plants.

Gene name	Species	R2R3-MYB number	MYB-related number	R1R2R3-MYB and atypical MYB number	Total number	Reference
*AtMYBs*	*Arabidopsis thaliana*	126	64	8	198	Chen et al., 2006
*OsMYBs*	*Oryza sativa*	148	87	4	239	Kang et al., 2022
*GmMYBs*	*Glycine max*	244	0	10	254	Du et al., 2012
*BvMYBs*	*Beta vulgaris*	70	0	5	75	Stracke et al., 2014
*SlMYBs*	*Solanum lycopersicum*	122	0	5	127	Li et al., 2016
*PhMYBs*	*Petunia hybrida*	106	40	9	155	Chen et al., 2021a
*AcMYBs*	*Actinidia chinensis*	91	87	3	181	Xia et al., 2022
*CaMYBs*	*Capsicum annuum*	116	92	7	215	Arce-Rodríguez et al., 2021
*MaMYBs*	*Musa acuminata*	222	73	10	305	Tan et al., 2020
*MbMYBs*	*Musa balbisiana*	184	59	8	251	Tan et al., 2020
*HuMYBs*	*Hylocereus undatus*	105	75	5	185	Xie et al., 2021a
*StMYBs*	*Solanum tuberosum*	124	90	3	217	Li et al., 2021
*RsMYBs*	*Raphanus sativus*	174	2	11	187	Muleke et al., 2021
*DoMYBs*	*Dendrobium officinale*	117	42	5	164	He et al., 2019
*AhMYBs*	*Arachis hypogaea*	209	219	15	443	Bertioli et al., 2019
*DlMYBs*	*Dimocarpus longan*	119	95	5	219	Chen et al., 2022a
*BnMYBs*	*Brassica napus*	429	227	24	680	Li et al., 2020a
*CeMYBs*	*Casuarina equisetifolia*	107	69	6	182	Wang et al., 2021b
*PaMYBs*	*Prunus avium*	14	51	4	69	Sabir et al., 2022

**Figure 4:**
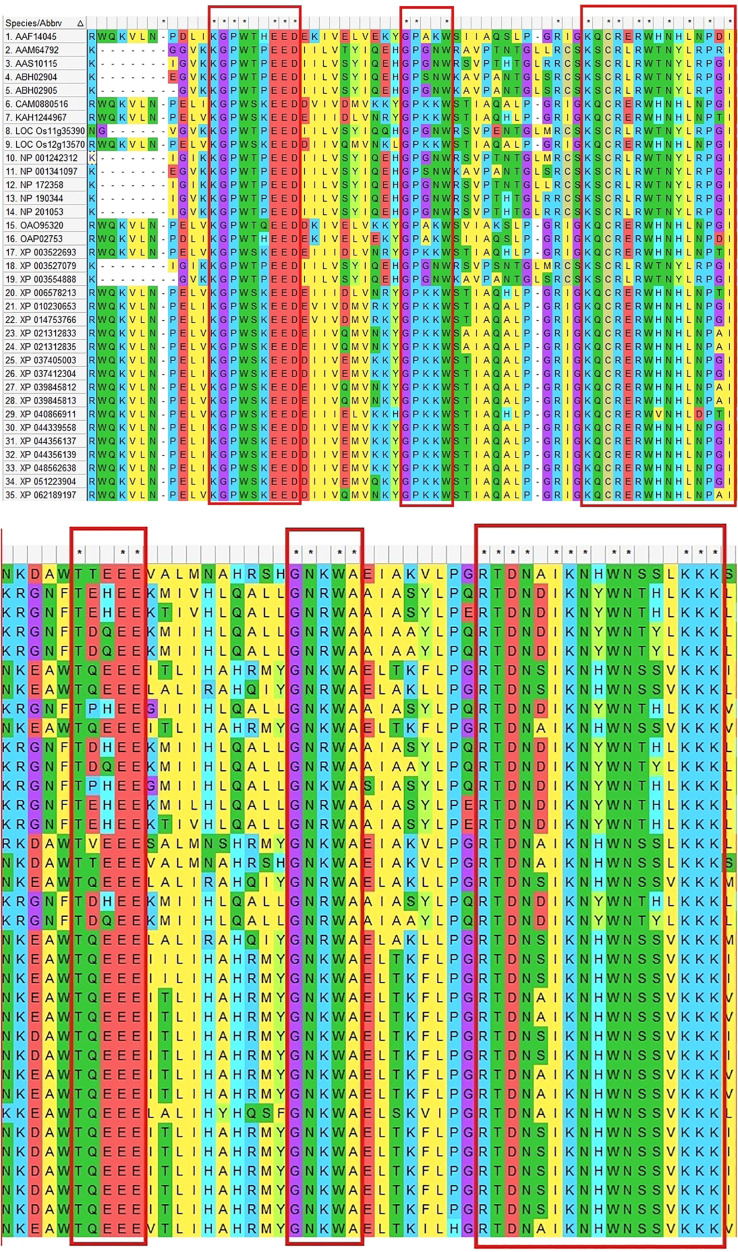
Conserved amino acid structural domains of MYB transcription factors in *Arabidopsis thaliana*, soybean and other species. The asterisk ‘*’ and red boxes indicate possible conserved structural domains in the figure.

By selecting *Arabidopsis thaliana* L., *Oryza sativa* and other species, a number of amino acid sequences were found in MYB family transcription factor proteins, and therefore it was hypothesized that these amino acid sequences might be MYB family transcription factors conserved amino acid sequences. As shown in the **Figure 4**, amino acid sequences such as KGPW**EED, GP**W, K*CR*RW*N*L*P*I, T**EE, GN*WA and RTDN*IKN*WN***KKK are conserved amino acid sequences of MYB family transcription factors (**Figure 4**). To investigate the phylogenetic and evolutionary relationships of MYB family transcription factors in different species, we constructed phylogenetic trees of the amino acid sequences of some MYB family transcription factors in *Arabidopsis thaliana*, *Oryza sativa* and *Glycine max Panicum virgatum, Phragmites australis* and *Sorghum bicolor.* using MEGA 11.0 software (Tamura et al., 2021). Among them, we found that GmMYB187 and GmMYB306 were highly related, GmMYB392 and GmMYB60 were highly related, AtMYB94 and AtMYB96 were highly related, OsMYB36a, OsMYB36b and OsMYB30 were highly related, which indicated that the MYB family of transcription factors were highly related in various species have close affinities between them. At the same time, we also found that MYB transcription factors between different species have strong phylogenetic relationships. Such as, AtMYB94 and GmMYB60 were highly related, AtMYB60 and OsMYB4P were highly related, AtMYB3R-5, GmMYB3R-1 isoform X1 and OsMYB3R2-L were highly related, it is possible that WRKY family transcription factors have similar functions in different species. MYB transcription factors are involved in regulating plant growth and development, secondary metabolism, and response to environmental stress (**Supplementary Figure S1**).

### 1.2 Transcriptional regulation mechanism of MYB transcription factor on target genes

MYB transcription factors have a conserved DNA binding domain in their structure. These transcription factors are composed of three conserved functional domains, a DNA binding domain (DBD), a transcription activation domain (TAD) and an incompletely defined negative regulatory domain (NRD) (Thompson and Ramsay, 1995). Ogata et al. believe that R2 and R3 are necessary for MYB transcription factor to recognize DNA sequences, and the C-terminal helix of R3 subunit can specifically bind to the core sequence in the cis acting element (Ogata et al., 1995). The cis acting elements that can be recognized by MYB transcription factors are called MBS (MYB-binding sites), which are generally rich in adenine and cytosine residues, such as (T/C)AAC(G/T) G(A/C/T)(A/C/T), (C/T)NGTT(A/G), ACC(A/T)A(A/C)(T/C), ACC (A/T)(A/C/T)(A/C/T) (Prouse and Campbell, 2012).

The sequence (T/C)AAC(G/T)G(A/C/T)(A/C/T) widely exists in the promoters of various stress response genes. Shukla et al. found that SbMYB44 can combine with the TAACTG motif on the promoters of many stress response genes to activate the expression of related genes (Shukla et al., 2015). Yang et al. found that OsMYB5P can regulate gene transcription and expression by recognizing and combining the CAACTG motif on the downstream target gene *OsPT5* promoter (Yang et al., 2018).

MYB transcription factors can also bind to the (C/T)NGTT(A/G) sequence and participate in regulating the biosynthesis of certain secondary metabolites. Zhou et al. found that FtMYB11 can directly bind to the AATAGTT sequence in its target gene promoter region, inhibiting the biosynthesis of phenylpropanoids in *Fagopyrum tataricum* (Zhou et al., 2017).

The sequence ACC(A/T)A(A/C)(T/C) and ACC(A/T)(A/C/T)(A/C/T) are known as AC-box, in which ACC is the core identification sequence. Studies have shown that both PtMYB4 and EgMYB2 can bind to AC-box to regulate the biosynthesis of lignin (Patzlaff et al., 2003). In addition, some MYB transcription factors in plants can also interact with E-box (CANNTG) and I-box (GATAAG). For example, LeMYBI can bind with I-box (Rose et al., 1999). BplMYB46 can bind with E-box (CANNTG), TC box (T(G/A)TCG (C/G)) and GT box (A(G/T)T(A/C)GT(T/G)C) (Guo et al., 2018).

## 2 Response of MYB transcription factor to plant hormones

**Figure 5:**
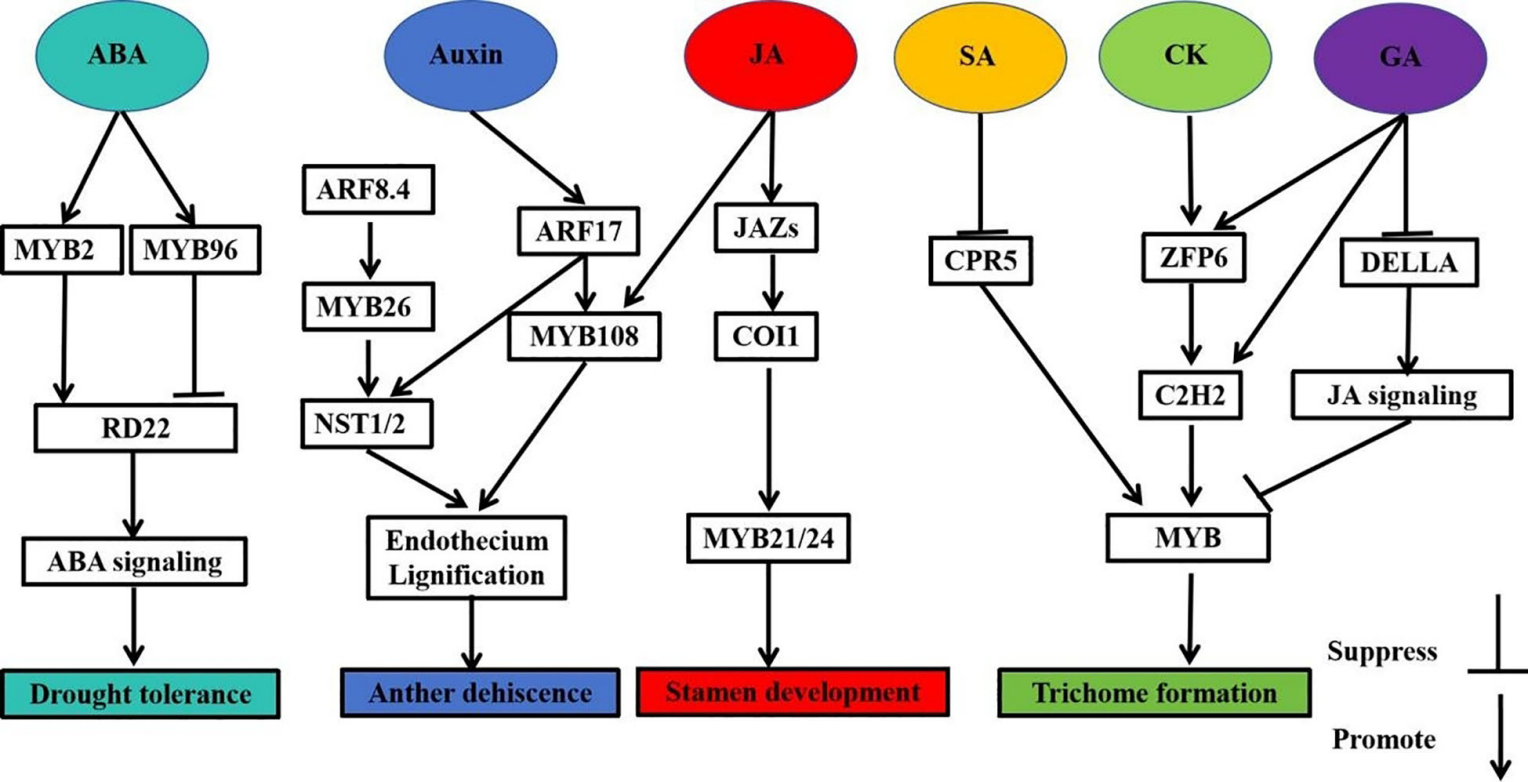
Integrated network diagram of MYB transcription factors involved in plant hormone regulation of growth and development as well as abiotic stresses. MYB: MYB transcription factors, RD22: responsive to dehydration 22, ARF8.4: auxin response factor 8.4, ARF17, auxin response factor 17; NST1/NST2, NAC secondary wall thickening promoting factor 1/2, JAZs, Jasmonate-ZIM domain containing protein; COI1, coronatine insensitive 1; CPR5, cell progression regulator 5; ZFP6, zinc finger protein 6; C2H2, C2H2 zinc finger protein; DELLA, negative regulatory factors in gibberellin signaling pathway.

Plant hormones are essential substances for regulating various physiological and biochemical reactions in plants to enable normal life activities. Previous studies have shown that MYB transcription factors can promote plant growth and development and response to abiotic stress by participating in plant hormone metabolism. For example, abscisic acid (ABA), auxin, jasmonic acid (JA), brassinosteroid (BR), salicylic acid (SA), gibberellin (GA) and auxin (IAA) (**Figure 5**; **Table 2**) (Li et al., 2020c; He et al., 2020; Yang et al., 2020a; Gao et al., 2018; Li et al., 2019a).

**Table 2:** Abiotic stress and plant growth and development responsive MYB transcription factors by plant hormone in plants.

Changed characteristic	Species	MYB transcription factors	Gene subfamily	Phytohormone	Reference
Drought	*Salicornia*	SbMYB15	R2R3-MYB	ABA	Joseph et al., 2013
Drought	*Fagopyrum tataricum*	FtMYB9	R2R3-MYB	ABA	Gao et al., 2017
Drought	*Fagopyrum tataricum*	FtMYB10	R2R3-MYB	ABA	Gao et al., 2016
Drought	*Fagopyrum tataricum*	FtMYB13	R2R3-MYB	ABA	Huang et al., 2018
Drought	*Gossypium arboreum Linn*	GaMYB85	R2R3-MYB	ABA	Butt et al., 2017
Drought	*Populus trichocarpa*	PtrMYB94	R2R3-MYB	ABA	Fang et al., 2020
Drought	Hordeum vulgare L.	HvMYB1	R2R3-MYB	ABA	Alexander et al., 2019
Drought	Triticum aestivum L.	TaP1MP1	R2R3-MYB	ABA	Liu et al., 2011
Cold	Oryza sativa L.	OsMYB30	R2R3-MYB	JA	Lv et al., 2017
Cold	*Manihot esculenta*	MeMYB2	R2R3-MYB	ABA	Ruan et al., 2017
Cold	*Fagopyrum tataricum*	FtMYB3	R2R3-MYB	SA and JA	Wang et al., 2022c
Heat	Pennisetum glaucum L.	PgMYB151	R2R3-MYB	ABA	Chanwala et al., 2023
Heat	Arabidopsis thaliana L.	AtMYBS1	–	SL	Li et al., 2023b
Salt	*Gossypium hirsutum*	GhMYB73	R2R3-MYB	ABA	Zhao et al., 2019
Salt	Arabidopsis thaliana L.	AtDIV2	MYB-related	ABA	Fang et al., 2018
Salt	Dianthus superbus L.	MdMYB23	R2R3-MYB	SA	Zheng et al., 2018
Salt	*Glycine soja Siebold & Zucc*	GsMYB15	R2R3-MYB	SA and JA	Shen et al., 2018
Nutrition	Arabidopsis thaliana L.	AtMYB62	R2R3-MYB	GA	Devaiah et al., 2009
Nutrition	Setaria italica L.	SiMYB42	R2R3-MYB	ABA	Qian et al., 2018
Heavy metals	Arabidopsis thaliana L.	AtMYB49	R2R3-MYB	ABA	Zhang et al., 2019a
Heavy metals	*Daucus carota*	DcMYB62	–	ABA	Sun et al., 2024
plant growth and development	Oryza sativa L.	OsCSA	R2R3-MYB	BR	Zhu X. et al., 2015
plant growth and development	Solanum tuberosum L.	StMYB60	R2R3-MYB	GA3, IAA	Sun et al., 2019
plant growth and development	*Rosa hybrida*	RhMYB108	R2R3-MYB	JA	Zhang et al., 2019b

Many studies have shown that the expression of MYB transcription factor gene in plants is induced by plant hormones. In 1997, the first hormone induced MYB transcription factors gene was found in *Arabidopsis thaliana*, namely *AtMYB2* gene. The expression of this gene is closely related to ABA signal transduction and is mainly involved in plant drought resistance response (Abe et al., 1997). Since then, more studies have found that a large number of MYB TF genes are regulated by hormone signals. At present, the plant hormone pathway with the most clear regulatory mechanism is ABA induced MYB TF gene expression. The regulation of MYB TFs on ABA can be divided into three types: dependent types, inducible types and mediated types. Dependent types: Park et al. found that MYB52, MYB70, MYB73 and MYB52 were involved in the stress response of *Arabidopsis thaliana* by regulating ABA dependent pathway (Park et al., 2011). Inducible types: Cai and Lee et al. found that MYB1, MYB2, MYB3R1 in wheat, which participate in plant response to stress conditions by regulating ABA accumulation (Cai et al., 2011; Lee et al., 2007). Mediated types: Cominelli et al. found that MYB44, MYB60, MYB13, MYB15 and MYB96 can change the size of stomata and improve the tolerance to drought environment by regulating the accumulation of ABA in *Arabidopsis thaliana* (Cominelli et al., 2005). ABA is a plant hormone that can be involved in a wide range of physiological processes in plants. Under drought stress, plants rapidly accumulate ABA. Xie et al. found that the MYB family transcription factors, MdMYB88 and MdMYB124, are essential for ABA accumulation in *Malus x domestics* after drought, and that MdMYB88 and MdMYB124 positively regulate water transpiration, photosynthetic capacity and stress tolerance in apple leaves under drought conditions. MdMYB88 and MdMYB124 also regulate ABA synthesis and catabolism genes and the expression of drought and ABA response genes (Xie et al., 2021b). Yuan et al. found that the expression of *PsMYB306*, a MYB transcription factor gene, is positively correlated with the expression of *9-CIS-EPOXYCAROTENOID DIOXYGENASE (PsNCED3)*, and that ABA increased the transcription of PsMYB306. Overexpression of *PsMYB306* in *Paeonia suffruticosa* inhibited seed germination and plant growth, and resulted in an increase ABA and a decrease in gibberellin (GA_1_ and GA_3_). PsMYB306 can negatively regulate the release of cold-induced bud dormancy by regulating the production of ABA (Yuan et al., 2024).

In addition to ABA, phytohormone JA regulation processes also involve MYB transcription factors. The phytohormone JA is an endogenous growth regulator present in higher plants. It induces stomatal closure, affects the uptake of N and P and the transport of organic matter such as glucose, and is closely related to plant resistance (Raza et al., 2022; Ruan et al., 2019; Wasternack and Song, 2017). Li et al. found that the MYB transcription factor can positively or negatively regulate anthocyanin biosynthesis. The MYB transcription factor mediates the JA signaling pathway during anthocyanin biosynthesis (Li et al., 2022a). Li et al. isolated that a novel R2R3-type MYB transcription factor GhODO1 is from *Gossypium hirsutum*, which plays an active role in resistance to *Verticillium dahliae*. The GhODO1 protein interacts with the promoters of the genes involved in lignin biosynthesis. The GhODO1 protein interacts with the promoters of the lignin biosynthesis-related genes *Gh4CL1* and *GhCAD3*, and GhODO1 is able to directly activate the expression of both genes and promote overall lignin accumulation. Furthermore, knockdown of *GhODO1* impaired JA-mediated defense signaling and JA accumulation (Zhu et al., 2022).

The phytohormone BR is a class of highly physiologically active steroid hormones that play important roles in plant growth and development, including stem and leaf growth, root growth and vascular tissue differentiation. It also plays an important role in plant defense against environmental stresses (Nolan et al., 2023; Yang et al., 2023; Tian et al., 2024). Peng et al. found that compared with the wild type, JA had a weaker induction effect on the “late” anthocyanin synthesis genes *DFR*, *LDOX* and *UF3GT* in BR mutant. In addition, the expression levels of MYB/bHLH transcription factors *PAP1*, *PAP2* and *GL3* induced by JA in BR mutant were lower than wild type. These transcription factors were components of WD-repeat/Myb/bHLH transcription complex, and mediated “late” anthocyanin biosynthesis genes (Peng et al., 2011). BR signals through the BES1/BZR1 (*bri1-ethylmethane sulfonate repressor 1/canola azole resistance 1*) family of transcription factors. And a direct target gene of *BES1*, the MYB transcription factor AtMYB30, was identified by Li et al. The *Atmyb30* mutant shows a reduced response to BR and enhances the dwarfing phenotype of a weak allele of the BR receptor mutant *bri1*. Many BR-regulated genes showed reduced expression and/or hormone induction in *Atmyb30* mutants, suggesting that AtMYB30 promotes the expression of a subset of BR target genes. AtMYB30 and BES1 bind to conserved MYB binding sites and E-box sequences in the promoters of BR and AtMYB30 regulated genes, respectively (Li et al., 2009). Chen et al. found that the dehydration-induced *GmMYB14* gene plays a role in the regulation of soybean architecture, high-density yield and drought tolerance through the BR pathway. The endogenous BR content of *GmMYB14-OX* plants was reduced, whereas exogenous application of BR partially rescued the phenotype of *GmMYB14-OX* plants. In addition, GmMYB14 was found to bind directly to the promoter of *GmBEN1* and up-regulate its expression, leading to a reduction in BR content in *GmMYB14-OX* plants. Drought tolerance of *GmMYB14-OX* plants was also improved under field conditions (Chen et al., 2021b).

Studies have shown that when active SA is applied externally, the expression of MYB transcription factors will increase in tobacco and the content of disease resistance related proteins will also increase accordingly (Yang and Klessig, 1996). The phytohormone SA can improve plant resistance to cold and drought. When plants are subjected to stress, some are injured to the point of death and others survive, although their physiological activities are affected to varying degrees (Janda et al., 2020; Frerigmann and Gigolashvili, 2014). The R2R3 MYB transcription factor GhMYB18 is involved in the defense response against the cotton aphid by participating in the synthesis of SA and flavonoids. GhMYB18 was identified as a gene that is up-regulated in upland *Gossypium hirsutum* L. plants attacked by *Aphis gossypii Glover*. Transient overexpression of *GhMYB18* in cotton activated the SA and phenylpropane signaling pathways and promoted the synthesis of SA and flavonoids, thereby enhancing tolerance to cotton aphid feeding, and vice versa. GhMYB18 also significantly increased the activities of defense-related enzymes, including catalase (CAT), peroxidase (POD), polyphenol oxidase (PPO) and phenylalanine deaminase (PAL) (Hu et al., 2023). Pyrethrins are terpene mixtures with insecticidal properties that accumulate in the above-ground parts of the pyrethrum (*Tanacetum cinerariifolium*). Zhou et al. reported the isolation and characterization of the *Tanacetum cinerariifolium* MYB transcription factor gene, which encodes the R3 MYB protein TcMYB8 with a large number of hormone-responsive elements in its promoter. Expression of the *TcMYB8* gene tended to decrease during flower and leaf development and was induced by JA, SA and ABA. Transient overexpression of *TcMYB8* increased the expression of the key enzyme genes *TcCHS* and *TcGLIP*, and increased pyrethrin levels. Further analysis revealed that TcMYB8 can directly bind cis-elements in *proTcCHS* and *proTcGLIP* and activate their expression, thereby regulating pyrethrin biosynthesis (Zhou et al., 2022).

In summary, most plant hormones can be regulated by MYB transcription factors, which shows that MYB transcription factors are closely related to plant hormone metabolism. Complex synergistic or antagonistic networks regulate the activities of different hormones in plants. MYB transcription factor genes have been implicated in ABA, JA, BR, SA and other pathways. Whether MYB transcription factor gene is involved in other hormone pathways and whether there is a deeper regulatory network between them remain to be answered.

## 3 Mode of action of MYB transcription factors in plants

As plants grow and develop, they are able to respond to changing external conditions in a timely manner (Tolosa and Zhang, 2020). Plants adjust their adaptation strategies in a timely manner according to changes in the external environment during their growth and development process. MYB regulates the homeostasis balance and tolerance to abiotic stress in plant cells at the transcriptional level. Its mode of action includes directly acting on the transcriptional regulatory sites of downstream response target genes after activation, or transmitting signals after interacting with upstream and downstream factors, and integrating multiple signaling pathways to respond to abiotic stress (Roy, 2016; Li et al., 2019b; Baillo et al., 2019).

### 3.1 Interaction between MYB and upstream and downstream factors

MYB mainly activates or inhibits transcription by directly binding to specific DNA sequences in downstream target gene promoter regions. AtMYB73 directly binds to the promoter region of the actin depolymerizing factors (ADF) gene, inhibiting its expression and hindering actin depolymerization in *Arabidopsis thaliana*, thereby altering the composition of the plant cytoskeleton (Wang et al., 2021c). Zhou et al. Identified that the CgMYB1 transcription factors, a member of the R2R3-MYB TF family. The *CgMYB1* transcription factors gene is induced by salt and cold stresses. Overexpression of *CgMYB1* in *Arabidopsis* significantly enhanced salt and cold tolerance. The interaction between CgMYB1 and the promoter of *CgbHLH001*, followed by the activation of the downstream stress-responsive genes, mediates the stress tolerance and improves the survival under salt and cold stress (Zhou et al., 2023b). Overexpression of the REVEILLE-8-type transcription factor *CstMYB1R1* in *Crocus floral* was explored for its possible role in regulating crocus flavonoid and anthocyanin biosynthetic pathway. The yeast one-hybrid technique was used to verify that CstMYB1R1 interacts with the promoter of the *LDOX* gene to directly regulate its transcription. The expression of *CstMYB1R1* significantly increased the levels of flavonoids and anthocyanins in *Nicotiana tabacum* and improved the abiotic stress tolerance of the plants (Bhat et al., 2023). Du et al. Identified that the N-terminal domain of MYB transcription factor MdMYB108L, which was significantly induced under salt stress, as transcriptionally active. Overexpression of *MdMYB108L* increased the germination rate, the length of the primary root and the antioxidant activities of catalase and peroxidase in transgenic *Arabidopsis* seeds and reduced the accumulation of reactive oxygen species (ROS). The overexpression of *MdMYB108L* also increased the photosynthetic capacity of the hairy root tissue (leaves) under salt stress. MdMYB108L was capable of binding to the *MdNHX1* promoter and positively regulating the transcription of the apple salt tolerance gene *MdNHX1*, thereby enhancing the salt tolerance of the transgenic plants (Du et al., 2022). According to the review, MYB transcription factor directly binds to the cis acting elements in the promoter regions of downstream target genes, regulates their transcription levels, and thereby regulates plant growth and development as well as transmits environmental signals.

Furthermore, the binding of MYB transcription factor to downstream target gene promoter sequences is also influenced by upstream protein interactions, thereby regulating changes in plant phenotype. Under ultraviolet radiation, AtMYB73/77 interacts with photoreceptors, inhibiting the binding activity of MYB73/77 to downstream auxin responsive target genes and negatively regulating hypocotyl elongation and lateral root development in *Arabidopsis thaliana* (Yang et al., 2020c). The N-terminus of the R3 repeat sequence of the MYB transcription factor contains a bHLH structural domain that binds to bHLH and WD40 to form the MBW complex, and flavonoid MYB deterrents are able to bind to basic helix-loop-helix factors and disrupt the MBW complex. For example, AtMYB75/90/113 associates with bHLHs (GL3, EGL3 and TT8) and WD40 (TTG1) to form the MBW complex in *Arabidopsis*, which controls the expression of the downstream anthocyanin late biosynthetic genes LDOX and DFR (Ma et al., 2019; Zheng et al., 2019). Li et al. found the expression patterns of *FhPAP1* in *Freesia hybrida* and the late synthesis genes involved in anthocyanin synthesis are similar. FhPAP1 can not only activate the expression of structural genes in the anthocyanin synthesis pathway, but also activate endogenous bHLH2 transcription factor genes in plants, such as FhTT8L in *Freesia hybrida*, AtTT8 in *Arabidopsis*, and NtAN1 in *Nicotiana tabacum*. In addition, FhPAP1 can also interact with bHLH transcription factor FhTT8L and WD40 protein FhTTG1 to form MBW complexes (Li et al., 2020d). Li et al. discovered that FhMYB27 and FhMYBx have different regulatory mechanisms: FhMYB27 mainly interacts with the MBW complex member FhTT8L, and then binds to the MBW complex in the form of a cofactor. By utilizing its strong transcriptional inhibitory activity, it transforms the MBW complex that originally had a positive regulatory effect into an MBW complex with inhibitory activity, thereby inhibiting the expression of downstream genes (Li et al., 2020e). Members of the MYB family can also bind to each other to form multimers. Such as, BplMYB46 can heterodimerize with BplMYB6, 8, 11, 12 and 13 to enhance binding to downstream target genes in *Betula platyphylla*. When *BplMYB46* and *BplMYB13* were coexpressed, the heterodimer formed by them enhanced the ROS scavenging ability by increasing the transcription of downstream genes encoding superoxide dismutase (SOD), POD and glutathione sulfotransferase (Wang et al., 2019b). Thus, MYB transcription factors can activate or repress downstream gene expression after interacting with upstream factors, thereby influencing downstream gene expression and regulating plant tolerance to abiotic stress.

## 4 Role of MYB transcription factors in response to abiotic stresses

Abiotic stresses include drought, salt, high temperature, low temperature, nutrients, heavy metals (Zhang et al., 2022b; Habibpourmehraban et al., 2023). They are the major abiotic stress factors which have an impact on plant growth and development, crop yield and crop quality. Abiotic stresses can severely impede the uptake of soil nutrients and water by plants, leading to water loss, stomatal closure, which affects plant photosynthesis, growth inhibition, metabolic disorders, accelerated senescence, which severely affects plant growth and can even lead to plant death (Dubos et al., 2010; Tong et al., 2024). Plants have evolved multiple ways to resist external abiotic stress. Indicating that MYB family transcription factors are widely involved in regulating plant responses to various abiotic stresses (Baldoni et al., 2015; Colombage et al., 2023).

### 4.1 MYB transcription factors in response to drought stress

Water is required for plant growth and development, and drought causes increased evaporation of water from plants, decreases soil water availability, and interferes with water transport processes in plants, resulting in irreversible damage to plants such as wilting of canopy leaves, branch dieback, and even death of the entire plant. The physiological mechanisms underlying drought-induced plant death are now the focus of intense research (Huang et al., 2021a; Vadez et al., 2024; Huang et al., 2021b; Chieb and Gachomo, 2023). Zhang et al. found that ZmMYB-CC10 improves drought tolerance in maize by reducing oxidative damage. ZmMYB-CC10 increases APX activity and decreases H_2_O_2_ levels. ZmMYB-CC10 was also shown to activate the expression of *ZmAPX4* by binding directly to its promoter using yeast one-hybrid crosses and luciferase assays (Zhang et al., 2022a). *CaDIM1* was identified by Lim et al. CaDIM1 has an N-terminal MYB structural domain and a C-terminal acidic domain, which are responsible for recognizing and activating target genes, respectively. *CaDIM1*-silenced plants exhibited ABA-insensitive and drought-sensitive phenotypes as well as reduced expression of adversity-responsive genes (Lim et al., 2022). The R2R3 MYB transcription factor MYB44-5A in *Triticum aestivum* L. was identified by Peng et al. The overexpression of *TaMYB44-5A* reduced the tolerance to drought and the sensitivity of transgenic *Arabidopsis thaliana* to ABA. At the same time, TaMYB44-5A down-regulated the expression levels of drought- and ABA-responsive genes, and TaMYB44-5A bound directly to the MYB binding site on the promoter and repressed the transcription level of *TaRD22-3A* (Peng et al., 2024). MYB transcription factor PtrMYB94 involved in drought response and ABA signaling in *Populus trichocarpa*, was identified by Fang et al. The overexpression of *PtrMYB94* improved the drought response of the plants. Seed germination was inhibited and ABA levels were significantly increased in overexpressing *PtrMYB94* plants. Overexpression of *PtrMYB94* plants also up-regulated the transcript levels of some ABA and drought-responsive genes *ABA1* and *DREB2* (Fang et al., 2020).

### 4.2 Molecular mechanisms of MYB transcription factors associated with salt stress

The visible symptoms of salt damage are the greening of the leaf tips, followed by the scorching, browning and death of the leaves. The result is stunted plant growth, poor root development, sterility and a reduction in the production of seeds. Salinity can cause soil crusting, poor soil structure, easy autodispersion of soil particles after irrigation and crusting, which in turn prevents water infiltration and reduces the soil’s water-holding capacity. This leads to reduced soil aeration and water conductivity, which severely affects plant growth and development and results in reduced yields (Zhou et al., 2024a, 2024b; Zhang et al., 2024a; Kausar and Komatsu, 2022; Xiao and Zhou, 2023). The MYB transcription factor MYB148 has been implicated in salt stress responses by Park et al. Salt and drought treatments increased *PagMYB148* expression in hybrid *Populus alba x P. glutulosa*. However, *pagmyb148* knockout plants exhibited a more sensitive phenotype under salt stress than wild-type plants. The chlorophyll content of the *pagmyb148* knockout plants was lower than that of the wild type under salt stress. The expression of genes involved in the salt stress response was higher in the *pagmyb148* knockout plants than in the wild type (Park et al., 2024). Wang et al. Identified SaR2R3-MYB15 transcriptional activity and nuclear localization. The overexpression of *SaR2R3-MYB15* increased the activity of antioxidant enzymes and the accumulation of proline, but decreased the level of malondialdehyde (MDA), which means that they have the potential to improve salt tolerance (Wang et al., 2024a). A total of 210 MYB transcription factor SbMYB1- SbMYB210 were identified by Lu et al. *SbMYBAS1* (*SbMYB119*) was found to be downregulated under salt stress conditions. Overexpression of *SbMYBAS1* in *Arabidopsis* plants had a significantly lower dry weight and chlorophyll content than the wild type under salt stress conditions, but a significantly higher membrane permeability, MDA content and Na^+^/K^+^ ratio than the wild type. Results also showed that SbMYBAS1 is capable of regulating expression of *AtGSTU17*, *AtGSTU16*, *AtP5CS2*, *AtUGT88A1*, *AtUGT85A2*, *AtOPR2* and *AtPCR2* under salt stress conditions (Lu et al., 2023). These results indicate that MYBs also play an important role in plant response to salt stress.

### 4.3 MYB transcription factors involved in plant response to temperature stress

Temperature is a key factor in plant growth and development. Temperature affects plant growth and ultimately crop yield, along with factors such as light, carbon dioxide, humidity, water and nutrient levels. Plants grow best when the temperature is kept just right for them to be able to grow. With both positive and negative effects, the higher the temperature, the faster most biological processes take place. For example, in most cases this can lead to a faster rate of growth and an increase in the yield of fruit crops (Huang et al., 2023; Wen et al., 2024; Islam et al., 2024).

#### 4.3.1 MYB transcription factors and high-temperature stress

However, the occurrence of respiration can have a negative effect as there will be less energy available for fruit development and the fruit will be smaller. Some of the effects are short term and some are long term. For example, the assimilative balance of a plant is affected by the temperature in an immediate way, whereas the formation of flowers is determined by the climatic conditions over a much longer period of time. The plant will increase its transpiration rate to cool down if the temperature is too high and This can cause the plant to lose more water and can lead to the death of the plants (Zhou et al., 2023a; Mariana et al., 2024; Zhu et al., 2023). Zhang et al. identified 174 MYB family members using a high-quality passion fruit genome: 98 2R-MYB, 5 3R-MYB, and 71 1R-MYB (MYB-relate). 10 representative PeMYB genes were selected for a quantitative verification of their expression levels. Most of the genes were differentially induced under cold, high temperature, drought and salt stress, with *PeMYB87* being significantly responsive to the expression induced by high temperature and to the overexpression of the *PeMYB87* gene in the yeast system (Zhang et al., 2023). The mechanism of color change in purple chrysanthemum under high temperature stress was investigated by Li et al. The main anthocyanins were significantly down-regulated in the heat sensitive cultivars under high temperature conditions. Differences in the expression of the *CHS*, *DFR*, *ANS*, *GT1*, *3AT* and *UGT75C1* genes during the synthesis of anthocyanins were found in heat-sensitive and heat-tolerant cultivars. Genes that were significantly negatively correlated with down-regulation of anthocyanin content included two MYB transcription factor genes, *Cse_sc001798.1_g020.1* and *Cse_sc006944.1_g010.1*, which can regulate anthocyanin accumulation in chrysanthemums under high-temperature stress (Li et al., 2024). Dragon fruit (*Hylocereus polyrhizus*) was found to be highly resistant to high temperature and drought stress by Xiao et al. *HpMYB72*, that is differentially expressed under high temperature in *Hylocereus polyrhizus*, and ectopic overexpression of *HpMYB72* in *Arabidopsis thaliana* improved the growth performance under high temperature stress and increased the germination rate. Oxidative damage was ameliorated by reducing the accumulation of ROS under high temperature stress. At the same time, the level of osmoregulatory substances was increased, thereby reducing the water loss caused by high temperature (Xiao et al., 2024b). These results indicate that MYBs also play an important role in plant response to high temperature stress.

#### 4.3.2 MYB transcription factors and low-temperature stress

When plants are exposed to low temperatures, the rate of photosynthesis decreases significantly. This is because low temperatures cause damage to the photosynthetic system and reduce the activity of photosynthetic enzymes such as PEP carboxylase (PEPcase). In addition, the plasma membrane of the cell becomes porous or cracked, which greatly increases the permeability of the plasma membrane and allows free diffusion of ions or soluble substances to the outside. This slows down the transport and transformation of photosynthetic products, thus slowing down plant growth (Tiwari et al., 2023; Xiao et al., 2024a). Weng et al. identified an *Arabidopsis* gain-of-function mutant, *ROC1(S)(58F)*, with enhanced cold tolerance and enhanced JA and oxidative stress-responsive gene expression. JA biosynthesis genes (*AtAOC1* and *AtOPR3*) and signaling genes (*AtJAZ5*, *AtJAZ10* and *AtMYB15*) were down-regulated in the mutant. The transcripts and activities of ROS scavenging enzymes (SOD/POD/MDHAR) were increased in mutants subjected to cold stress, probably due to the alleviation of ROS-induced oxidative stress, which contributes to the freezing tolerance of the mutants (Weng et al., 2020). Yang et al. isolated a cold-inducible MYB transcription factor DgMYB2 from chrysanthemum (*Chrysanthemum morifolium Ramat*). Overexpression of *DgMYB2* increased the cold tolerance of chrysanthemum, whereas antisense suppression lines showed a reduced cold tolerance compared with the wild type. Meanwhile, DgMYB2 directly targeted *DgGPX1* and increased glutathione peroxidase activity to reduce the accumulation of reactive oxygen species, which improved chrysanthemum cold resistance (Yang et al., 2022c). Li et al. obtained a novel 1R MYB transcription factor gene from the diploid strawberry by cloning and named it FvMYB114. Overexpression of *FvMYB114* greatly enhanced the adaptation and tolerance of *Arabidopsis* to salt and low temperature. Proline and chlorophyll contents as well as SOD, POD and CAT activities of the transgenic plants were higher than those of wild-type and unloaded *Arabidopsis* lines under salt and low temperature stress. However, the wild type and unloaded lines had higher levels of MDA. FvMYB114 also promoted the expression of the low temperature stress-related genes *AtCCA1*, *AtCOR4* and *AtCBF1/3* (Li et al., 2023a). Chen et al. analyze the relative expression of the MYB transcription factors *StMYB113* and *StMYB308* during different periods of low-temperature treatment. *StMYB113* and *StMYB308* could be expressed in response to low temperature and could promote anthocyanin synthesis. The study showed that StMYB113, which lacked the complete MYB structural domain, could not promote the accumulation of anthocyanins in *Nicotiana tabacum*, while StMYB308 could significantly promote the accumulation of anthocyanins (Chen et al., 2024). Chen et al. obtained an MYB TF AhMYB30 from peanut using a transgenic approach. Overexpression of *AhMYB30* increased the resistance of transgenic plants to freezing and salt stress in *Arabidopsis thaliana*. Expression of the stress response genes *RD29A* (*Response-to-Dehydration 29A*), *COR15A* (*Cold-Regulated 15A*), *KIN1* (*Kinesin 1*) and *ABI2* (*Abscisic acid Insensitive 2*) was increased in transgenic plants compared to wild type. It is therefore possible that AhMYB30 acts as a transcription factor in *Arabidopsis thaliana* to increase the tolerance of the plant to salinity and freezing (Chen et al., 2023a). These results indicate that MYBs also play an important role in plant response to low temperature stress.

### 4.4 Role of plant MYB transcription factors in response to nutritional element stress

Nitrogen (N), Phosphorus (P) and Potassium (K), which are essential plant nutrients, play extremely important physiological roles in helping plants grow and development (Kumar et al., 2021).

N is a component of the vitamin system and the energy system of the plant. The element nourishes leaves and helps branches, stalks, and stems (Antenozio et al., 2024). Cereal (*Setaria italica*) native to China, that is highly tolerant to low nutrient stress. Ge et al. systematically analyzed the cereal transcriptome under low nitrogen stress. There were 74 transcription factor genes in the differentially expressed genes (DEG), including 25 MYB-like transcription factors. Root development in *Arabidopsis* and overexpressing *SiMYB3* in *Oryza sativa* under low nitrogen stress was superior to that of the wild type. SiMYB3 could specifically bind to the MYB element in the promoter region of the *TAR2* promoter region of the growth hormone synthesis-related genes conserved in *Oryza sativa* and *Setaria italica*. SiMYB3 can regulate root development under low nitrogen conditions by regulating growth hormone synthesis in plant roots (Ge et al., 2019). Wang et al. identified all MYB genes in Phaeodactylum tricornutum, and analyzed the MYB transcription factor gene family at the genome level. The homology analysis of the MYB transcription factor genes indicated that PtMYB3, PtMYB15 and PtMYB21 can play important roles in regulating the circadian rhythm and response to nitrogen stress in *Phaeodactylum tricornutum* (Wang et al., 2022b).

**Figure 6:**
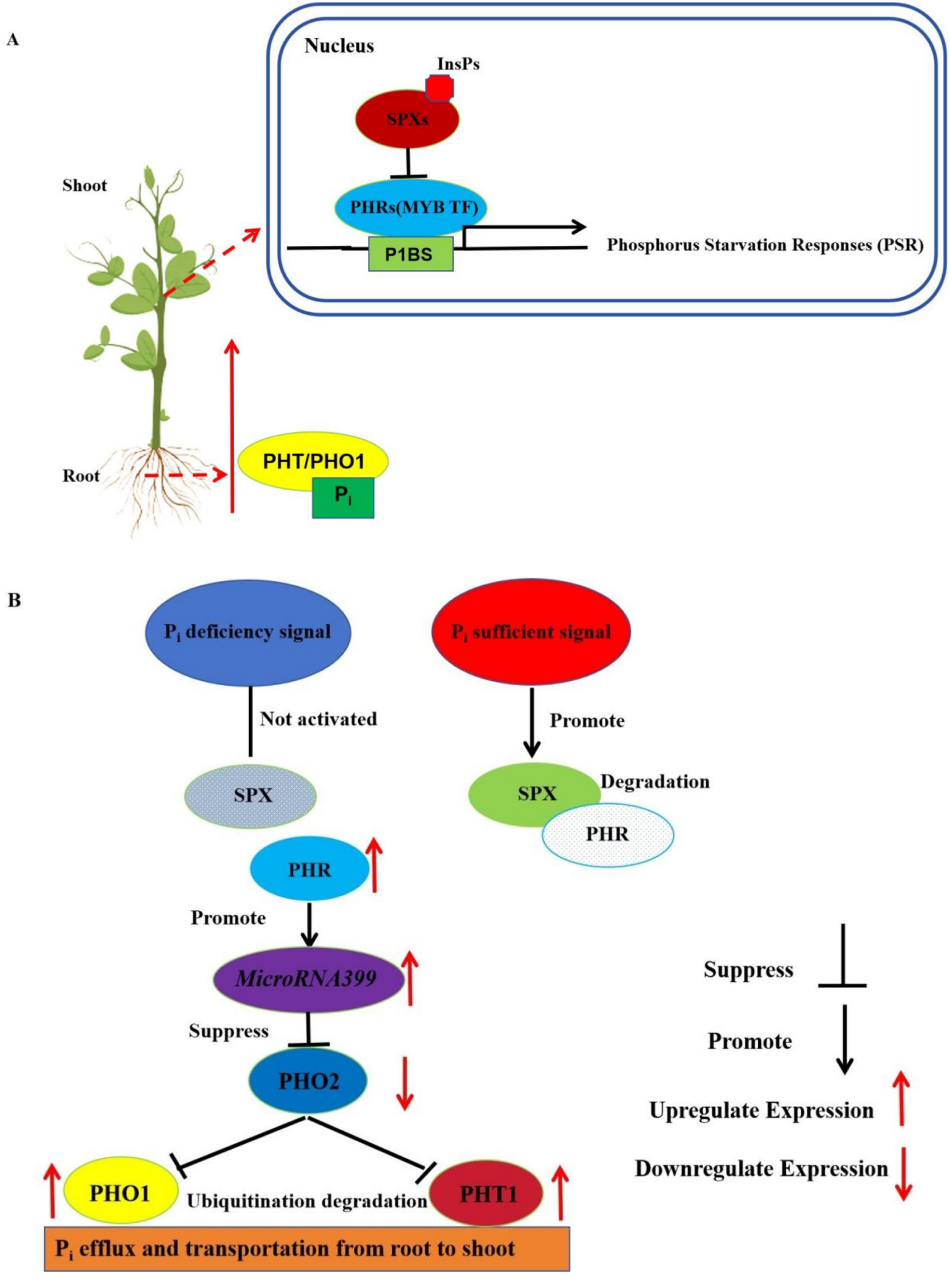
Schematic representation of the molecular regulation phosphorus homeostasis mediated through the MYB transcription factor PHR in plants. **(A)** Schematic representation of phosphate uptake and transport by plants through the root system. The red upward arrow represents the transport of Pi from the root to the shoot. Pi=phosphate. **(B)** Patterns of regulation of SPX proteins and PHR transcription factors when low and high phosphorus signaling differ. The red upward arrow represents increased expression. The red downward arrow represents decreased expression.

P determines the differentiation of flower buds, the development of pollen key elements, is the reproductive growth and nutritional growth of essential elements. At the same time, phosphorus is involved in various metabolisms in the body, including carbohydrate metabolism, promotion of nitrogen metabolism, and fat metabolism (Prathap et al., 2022). Plants take up phosphate from the soil mainly through phosphate transporter proteins (mainly PHT1 family proteins) in the root system and transport phosphate to the aboveground via transporter proteins such as PHO1 (Gu et al., 2016). PHR transcription factors, as MYB family transcription factors, can positively regulate the phosphorus deficiency response in plants, and it binds to the *P1BS* motif in the promoter region of phosphorus deficiency response genes, thus activating the expression of downstream genes. SPX proteins, as phosphorus receptors, can avoid toxicity caused by phosphorus over-accumulation by interacting with PHR1 (*Arabidopsis*) or PHR2 (*Oryza sativa*), and inhibiting their transcriptional activities under normal conditions, while SPX proteins do not affect PHR transcriptional activities when plants are in low phosphorus environments (Wang et al., 2014). It has been shown that SPX proteins do not directly sense phosphate, but instead sense soluble inositol polyphosphates (InsPs) (Wild et al., 2016). Inositol pyrophosphate InsP8 acts as an intracellular phosphate signaling substance to regulate phosphorus homeostasis by modulating the interaction of SPX1 with PHR1 (Dong et al., 2019). *Arabidopsis thaliana* accumulates InsP8 under phosphorus-sufficient conditions and promotes the binding of the InsP8-SPX complex to the CC structural domain of the PHR transcription factor, thereby repressing PHR-mediated phosphorus-deficiency-responsive gene expression (**Figures 6A, B**) (Ried et al., 2021). The *microRNA399* can play a key role in phosphorus homeostasis and phosphorus deficiency response through post-transcriptional regulation. *MicroRNA399* is induced to be expressed under phosphorus deficiency conditions and positively regulated by PHR1, which promotes phosphorus uptake and transport by inhibiting the mRNA expression of its target gene, the ubiquitin-conjugating E2 enzyme *PHO2*, and then increasing protein expression of PHO1 and PHT1 in the downstream of PHO2 (Liu et al., 2012; Xiao et al., 2022b). MiR399-PHO2 regulatory module also plays a similar role in other plants. For example, *miR399* is specifically induced by low phosphorus stress in maize, and overexpression of *miR399b* causes maize to overexploit phosphate in the shoot and develop symptoms of phosphorus toxicity (Du et al., 2018) (**Figure 6B**). Interestingly, miR399 also negatively regulates the expression of phosphate transport proteins *ZmPHT1;1*, *ZmPHT1;3* and *ZmPHT1;13* in maize, and this regulation is modulated by the long-chain non-coding RNA *PILNCR2*. Meanwhile, *PILNCR2* is induced by phosphorus deficiency and forms RNA/RNA dimers with ZmPHTs, thus interfering with the targeting of miR399 to *ZmPHTs* (Wang et al., 2023b).

The main function of K is to participate in plant metabolism, such as promoting photosynthesis and the transfer of photosynthesis products, regulating ion and water balance, promoting protein metabolism, and enhancing plant resistance (Mulet et al., 2023; Xiao et al., 2022a). Niu et al. found that RsMYB39 and RsMYB82 appeared to be non-canonical MYB anthocyanin activators and deterrents, respectively. It was confirmed that RsMYB39 strongly induced the promoter activity of the anthocyanin transport-related gene *RsGSTF12*, whereas RsMYB82 significantly reduced the expression of the anthocyanin biosynthesis gene *RsANS1*. Their data demonstrate the strong effect of potassium on sugar metabolism and signal transduction and its regulation of anthocyanin accumulation through different sugar signals and R2R3-MYB in a hierarchical regulatory system (Niu et al., 2023). These results indicate that MYB regulates the ability of plants to maintain normal growth and development under nutrient deficient conditions by enhancing the absorption capacity of plant roots for nutrients.

### 4.5 MYB transcription factors involved in plant response to heavy metals stress

Excessive amounts of heavy metals inhibit seed germination and seedling growth, damage antioxidant enzyme systems and membrane systems, and induce chromosomal aberrations. Appropriate metals can promote plant growth, but excessive heavy metals can form a greater toxicity to cells and affect plant growth and development (Zhang and Lu, 2024b; Liu et al., 2024a; Gu et al., 2016). Excessive cadmium (Cd) in soil poses a serious hazard to the survival and development of a wide range of organisms. Feng et al. identified a MYB family transcription factors PsMYB62 in *Potentilla sericea*. Net photosynthetic rate, stomatal conductance, transpiration rate, intercellular CO_2_ concentration, and chlorophyll content of *PsMYB62* overexpressing plants were significantly higher than that of the control after Cd treatment. The expression of *NtHMA3*, *NtYSL*, *NtPDR4* and *NtPDR5B* in the transgenic lines was significantly lower than that of the control, while the expression of *NtNAS3*, *NtSOD* and *NtGSH2* was significantly higher than that of the control (Feng et al., 2024). *Daucus carota* is a globally important root vegetable crop, and it has evolved multiple transcriptional regulatory mechanisms to cope with Cd stress. Sun et al. found that the expression level of *DcMYB62* was positively correlated with the accumulation pattern of carotenoids, and that the expression of *DcMYB62* improved Cd tolerance in *Arabidopsis* by increasing seed germination, root length, and overall survival. Heterologous expression of *DcMYB62* increased the transcription of genes associated with heavy metal resistance in *Arabidopsis*, particularly nicotinamide synthase (Sun et al., 2024). These results indicate that MYB also plays an important role in plant response to heavy metal stress.

## 5 MYB transcription factors are involved in the regulation of secondary metabolism in plants

MYB family transcription factors not only play important roles in regulating plant growth and development and abiotic stress response, but also participate in the regulation of plant primary and secondary metabolites. Among them, some important secondary metabolites, such as glucosides, flavonoids, terpenoids, lignans, and astragaloids. the MYB transcription factors are able to regulate their metabolic (Zhang et al., 2025; Klempnauer, 2024; Xie et al., 2016; Wasternack and Strnad, 2019).

### 5.1 MYB transcription factors are involved in glucosinolate metabolism in plants

Glucosinolate (GSL) is widely known as a secondary plant metabolite derived from amino acids and sugars. It functions not only as a protection against pests, but also helps plants to resist various diseases (Patil et al., 2024). Augustine et al. identified four MYB28 homologues BjMYB28-1, BjMYB28-2, BjMYB28-3, BjMYB28–4 from polyploid mustard (*Brassica juncea*), and phylogenetic analyses indicated that the four BjMYB28s proteins evolved through a process of hybridization and replication. The four BjMYB28s genes all encode functional MYB28 proteins and are involved in the synthesis of glucosinolates (Augustine et al., 2013). Seo et al. found in turnip (*B. rapa*) that the expression of some BrMYBs genes *BrMYB28*, *BrMYB34*, and *BrMYB51* are also increased under abiotic and biotic stress conditions. In addition, the function of three BrMYB28s transcription factor protein involved in the regulation of lipid, indole and aromatic GSL synthesis, respectively, as well as the expression of synthetic genes *BrAOP2* and *BrGSL-OH* in transgenic *B. rapa* was analyzed by *Agrobacterium* transformation (Seo et al., 2016). In addition, researchers have found that MYB transcription factor MYB28, MYB29 and MYB76 specific activation is involved in GSL synthesis. MYB transcription factor binding sites exist in the promoters of GSL biosynthetic genes, and MYB transcription factor can directly activate their expression (Hirai et al., 2007; Sønderby et al., 2010).

### 5.2 MYB transcription factors are involved in flavonoids metabolism in plants

**Figure 7:**
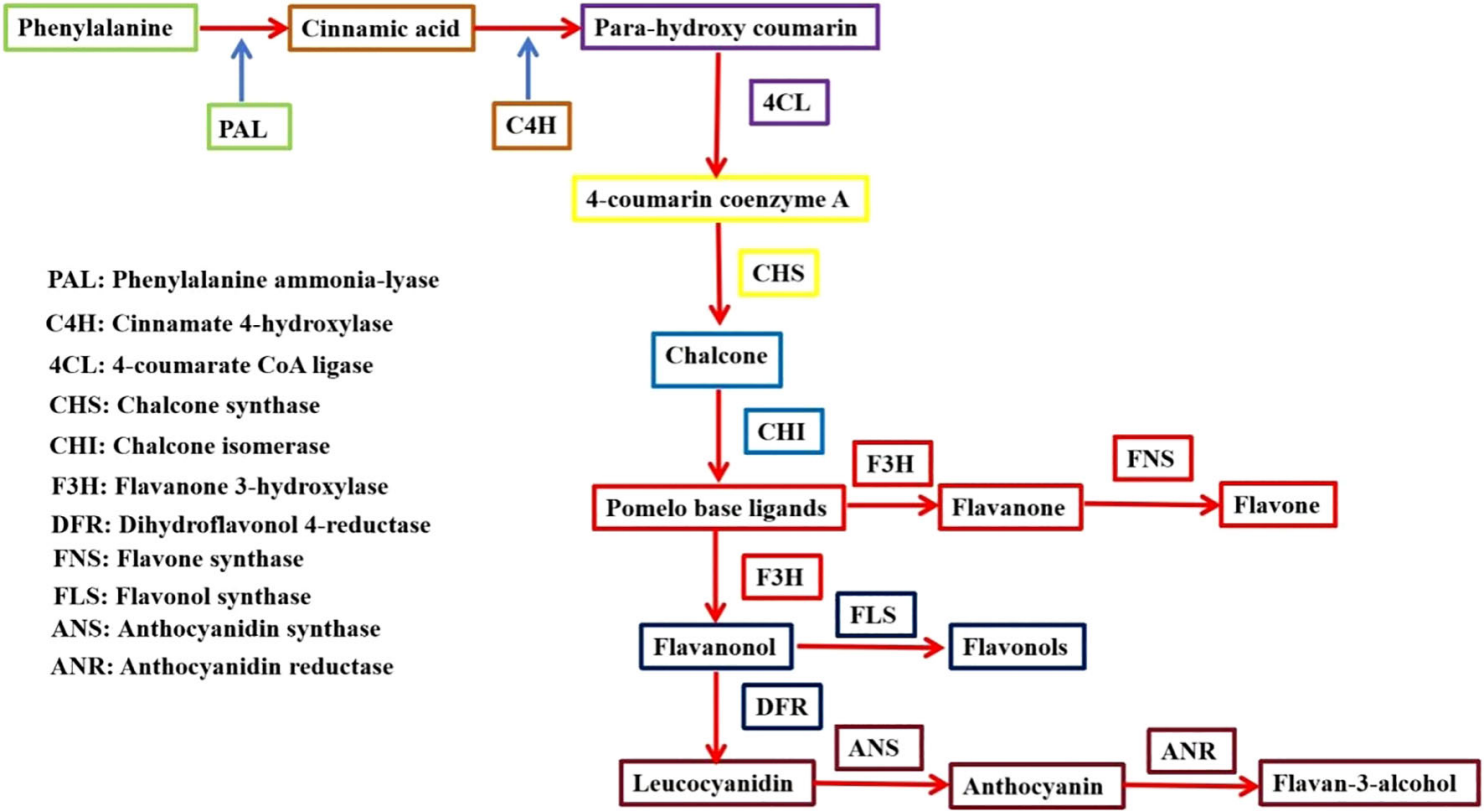
Flavonoid biosynthesis pathway. PAL, Phenylalanine ammonia-lyase; C4H, Cinnamate 4-hydroxylase; 4CL, 4-coumarate:CoA ligase; CHS, Chalcone synthase. CHI, Chalcone isomerase; F3H, Flavanone 3-hydroxylase; DFR, Dihydroflavonol 4-reductase; FNS, Flavone synthase; FLS, Flavonol synthase; ANS, Anthocyanidin Synthase; ANR, Anthocyanidin reductase.

Flavonoids are important secondary metabolites in plants, and anthocyanins, flavonols, and isoflavones are important components of them. As a class of multifunctional compounds, they include regulation of plant cell wall formation, resistance to UV-B damage and defense against diseases (Xu et al., 2014). However, the synthesis pathways of flavonoids are relatively numerous and complex, so we have briefly summarized a flowchart for a better understanding of flavonoid biosynthesis pathways (**Figure 7**). In addition to being regulated by enzymes involved in the synthetic pathway, the metabolic pathway is also regulated by the MYB family of transcription factors. By binding to structural genes in the plant body, MYB transcription factors are able to activate multiple related genes in the plant’s secondary metabolic synthesis pathway, causing them to be expressed synergistically, thereby initiating secondary metabolism in the plant.

Flavonoids have a wide variety of pharmacological activities and physiological functions, this has led to the widespread interest in this group of compounds, which is now the focus of research. Anthocyanins are a class of water-soluble pigments found widely in plants and determine the color of plant flowers, leaves, fruits, stems and seed coat. Anthocyanins also contribute to several physiological and biochemical processes, including attraction of insects for pollination, UV prevention, and stress protection in plants (Ahmed et al., 2015; Oh et al., 2011). Anthocyanin biosynthesis is transcriptionally regulated by the MYB-bHLH-WD (MBW) triad, and several MYB transcription factor proteins related to anthocyanin synthesis have been identified in plants, such as *Myrica rubra*, where MrMYB1 interacts with MrWD40–1 and MrbHLH1, to form the MBW complex that regulates anthocyanin accumulation (Niu et al., 2010). In *Populus*, PtrMYB57 can interact with bHLH131 and PtrTTG1 to negatively regulate anthocyanin biosynthesis (Wan et al., 2017). Other genes that perform similar functions include VvMYBC2L2 in *Vitis vinifera* (Zhu et al., 2018), CmMYB7 and CmMYB6 in *Chrysanthemum morifolium* (Xiang et al., 2019). Feng et al. cloned the R2R3-MYB transcription factor AgMYB2 from *Apium graveolens L.*, and heterologous expression of *AgMYB2* in *Arabidopsis thaliana* produced more anthocyanins and dark purple leaves and flowers. And a yeast two-hybrid assay experiment demonstrated the interaction between AgMYB2 and bHLH transcription factor proteins (Feng et al., 2018). Jiang et al. found an R2R3 MYB transcription factor, ThMYB14 in *T. hemsleyanum*. The overexpression *MYB14* or knockout of *myb14* lines significantly promoted or inhibited the accumulation of flavonoids, respectively. Promoter sequence analysis identified many potential MYB binding sites, including CCAAT box, MBS and MBSI elements upstream of flavonoid biosynthesis related genes. ThMYB14 promotes flavonoid accumulation by specifically recognizing AAC elements in these sites. In conclusion, ThMYB14 can regulate the accumulation of flavonoids in *T. hemsleyanum* (Jiang et al., 2025). Xu et al. identified a total of 26 R2R3-MYB TF in *T. hemsleyanum*, most of which were clustered into functional branches of abiotic stress. Through protein-protein interaction prediction, ThMYB4 and ThMYB7 related to flavonoid biosynthesis were screened out. ThMYB4 and ThMYB7 were positively correlated with genes of flavonoid biosynthesis pathway in *T. hemsleyanum*. The expression of *ThCHS* and *ThCHI* were significantly increased in hairy roots with overexpression of *ThMYB4* and *ThMYB7* lines, suggesting that ThMYB4 and ThMYB7 can act as regulators in flavonoid biosynthesis (Xu et al., 2023).

Flavonols are important flavonoids whose synthesis from dihydroflavonols is catalyzed by flavonol synthase (FLS). In *Arabidopsis*, AtMYB11, AtMYB12, and AtMYB111 are all able to independently activate the gene encoding chalcone synthase (CHS), and chalcone isomerase (CHI), flavanone 3-hydroxylase (F3H), and flavonol synthase (FLS) together determine flavonol content (Luo et al., 2008; Misra et al., 2010; Pandey et al., 2012). Matsui et al. identified a MYB transcription factor protein, FeMYBF1, from *Fagopyrum esculentum Moench* and performed amino acid sequence analysis and phylogenetic analyses, which indicated that expression of FeMYBF1 in the flavonol-deficient Arabidopsis triple mutant, *myb11/myb*12/*myb*111, promotes flavonol synthesis. Expression of *FeMYBF1* driven by the CaMV *35S* promoter in *Arabidopsis* resulted in up-regulation of *AtFLS1* expression and over-accumulation of flavonol glycosides (Matsui et al., 2018). The MYB transcription factor GtMYBP3 and GtMYBP4 in *Gentiana triflora* similarly activate the expression of flavonol synthesis genes and increase flavonol content when heterologously expressed in *Arabidopsis* (Nakatsuka et al., 2012). There are also some MYB transcription factors in other species, such as in *Pyrus betulifolia* PbMYB12b, in *Malus domestica* MdMYB22 can positively regulate flavonol biosynthesis (Zhai et al., 2019; Wang et al., 2017b). In *Glycine max* GmMYB176 regulates isoflavonoid biosynthesis by activating the expression of soybean chalcone synthase gene (Anguraj Vadivel et al., 2019). Isoflavonoids are a class of flavonoids found mainly in the Pteridophyceae family, which accumulate in the immature embryo of *Glycine max*. R1-MYB GmMYB176 is also involved in the regulation of CHS expression and isoflavone synthesis. Mutant *myb176* silencing leads to a decrease in the accumulation of isoflavonoids in hairy roots (Yi et al., 2010).

### 5.3 MYB transcription factors are involved in terpenoids metabolism in plants

Terpenoids are the most abundant class of plant natural products due to their extensive use in flavor, cosmetics, pharmaceuticals, agriculture and chemical industries. Therefore, there is a very broad development and application prospect (Bouvier et al., 2005). Reddy et al. identified the PGT-specific R2R3-MYB gene MsMYB in *Mentha* sp*icata Linn.* RNA-Seq data and functionally characterized it. To analysis of *MsMYB-RNAi* transgene lines showed an increase in monoterpene levels and *MsMYB* overexpression lines showed a decrease in monoterpene levels. The results suggest that MsMYB is a novel negative regulator of monoterpene biosynthesis. The study on the regulation of terpene metabolism by MYB transcription factors is still in the preliminary stage, and this is also the first report on the regulation of monoterpene synthesis by R2R3 MYB (Reddy et al., 2017).

### 5.4 MYB transcription factors are involved in lignins metabolism in plants

Lignin is one of the main components that make up the secondary walls of lignocells and fibers, which are synthesized via the phenylpropane pathway and are the main components that make up phytochelatins, which resistance to UV-B damage and pathogen attack (Boerjan et al., 2003). Three MYB transcription factors in *Arabidopsis*, AtMYB20, AtMYB42 and AtMYB43, activate lignin synthesis-related genes and mediate secondary wall formation, and silencing of these genes resulted in a significant reduction in lignin synthesis in *Arabidopsis* and led to defects in plant growth and development (Geng et al., 2020). CsMYB330 and CsMYB308 in *Citrus sinensis* have opposite regulatory effects, with the former activating and the latter inhibiting the lignification process (Jia et al., 2018). In *Eucalyptus* EgMYB1 specifically interacts with the histone variant EgH1.3, which strongly inhibits lignin deposition in the xylem cell wall. Thus preventing premature or inappropriate lignification of secondary walls (Soler et al., 2017). In addition, MuMYB31 also inhibits lignin synthesis in *Musa nana* (Tak et al., 2017). There are a number of other species with MYB family transcription factors that are also capable of regulating lignin synthesis, for example ZmMYB31, ZmMYB42 in *Zea mays*, PvMYB4a in *Panicumvirgatum*, LlMYB1 in *Leucaena leucocephala* and CmMYB1 in *Chrysanthemum morifolium* are able to inhibit lignin synthesis (Fornalé et al., 2010; Shen et al., 2012; Omer et al., 2013; Zhu et al., 2013).

### 5.5 MYB transcription factors are involved in other secondary metabolites in plants

Astragaloids compounds are low in normal tissues of the plant kingdom, and mono- and di-phenolic hydroxylated astragalus compounds are found mainly in the thin-walled cells of the xylem of plant tissues. When plants are infected by pathogens or externally stimulated, the total content of astragaloids in the stimulated tissue sites increases significantly. Therefore, natural astragaloids can be stress products of plants (Liang et al., 2023). Expression of *AtMYB14* and *AtMYB15* in grape trichome roots induces genes encoding stilbene synthases and leads to accumulation of glycosylated stilbenes (Höll et al., 2013). In *Solanum lycopersicum* epidermal trichome regulators Woolly and SlMYB31 are synergistically used in the biosynthesis of tomato cuticle waxes by regulating the expression of *SlCER6* (Xiong et al., 2020). In *Capsicum annuum* CaMYB31 can be a master regulator of capsaicinoid synthesis genes (Han et al., 2019). In *Salvia miltiorrhiza* SmMYB98 can regulate the biosynthesis of tanshinone and salvinorin in hairy roots (Hao et al., 2020). Thus the major regulatory role of MYB transcription factors in plant secondary metabolic processes can regulate the synthesis of a wide range of secondary metabolites.

### 5.6 MYB transcription factors are directly involved in the regulation of secondary metabolic biosynthesis

Some studies have found that MYB transcription factors regulate the synthesis of terpenoids mainly by directly regulating the transcriptional expression of structural genes in the terpenoid synthesis pathway. MYB transcription factors can positively regulate the expression of terpenoid synthesis pathway enzyme genes, thereby promoting the biosynthesis of terpenoids. Matías et al. found overexpressing *AaMYB1*in *Artemisia annua* can activate key enzymes in the artemisinin biosynthesis pathway, such as sophoride oxidase (*CYP71AV1*), sophoride synthase (*ADS*), farnesyl diphosphate synthase (*FDS*), artemisinin aldehydeΔ11 (13) reductase (*DBR2*), and aldehyde dehydrogenase (*ALDH1*) encoding genes, thereby increasing artemisinin synthesis. AaMYB1 activates the expression of enzymes *GA3ox1* and *GA3ox2* in the GA biosynthesis pathway, increasing GA synthesis and promoting the development of *Artemisia annua* glandular hairs. The density of glandular hairs is positively correlated with artemisinin content (Matías-Hernández et al., 2017). Although MYB transcription factor can positively regulate the biosynthesis of artemisinin, the mechanism is different. For example, MYB transcription factor AaBPF1 promotes the expression of enzyme genes of artemisinin biosynthesis pathway in *Artemisia annua*, while AaMIXTA1 increases the artemisinin content in *Artemisia annua* by increasing the growth density of glandular hairs (Ma et al., 2019; Shi et al., 2018). SmMYB98 plays a positive regulatory role in the biosynthesis of tanshinone and salvianolic acid in *Salvia miltiorrhiza*. SmMYB98 can directly bind to the promoters of the key enzyme genes (*SmGGPPS1*) in the terpene biosynthesis pathway and encoding phenylalanine ammonia lyase (*SmPAL1*) and encoding rosmarinic acid synthase (*SmRAS1*) in the phenolic acid biosynthesis pathway, directly regulate their expression levels and promote the synthesis of tanshinone and salvianolic acid (Hao et al., 2020).

In addition, MYB transcription factors can also negatively regulate the expression of terpenoid synthesis pathway enzyme genes, affecting the biosynthesis of terpenoid compounds. Reddy et al. found MsMYB can inhibit the expression of geranyl diphosphate synthase gene *GPPS* in *Mentha* sp*icata*, thereby reducing the accumulation of its catalytic product GPP *in vivo* and the reduction of monoterpene biosynthetic precursor GPP, which hinders the biosynthesis of limonene, carvone and other monoterpene substances (Reddy et al., 2017).

### 5.7 MYB cooperates with other transcription factors in the biosynthetic regulation of secondary metabolism biosynthesis

Some studies have found that MYB can not only play a regulatory role alone, but also form protein complexes with other transcription factors to participate in the regulation of secondary metabolic biosynthesis. FhMYB21L1 and FhMYB21L2 can significantly activate the expression of monoterpene synthase gene *FhTPS1* and promote the synthesis of linalool in *Freesia hybrida*. When FhMYC2 interacts with FhMYB21L2 to form a protein complex, it interferes with the binding of *FhTPS1* promoter, inhibits the expression of *FhTPS1* and inhibits the synthesis of linalool. The heterologous expression of *FhMYC2* and *FhMYB21L2* can also inhibit the expression of terpene synthase gene *AtTPS14* in *Arabidopsis thaliana*. This study demonstrated that MYB- bHLH protein complex was involved in the regulation of plant monoterpene biosynthesis (Yang et al., 2020b). TmMYB39 can interact with TmbHLH13 to form a TmMYB39-TmbHLH13 complex in *Taxus madia*, and significantly activate the expression of taxol biosynthesis genes *GGPPS* and *T46OH*, promoting the synthesis of taxol in *Taxus media* (Yu et al., 2022).

MYB transcription factors not only regulate terpene synthesis by forming complexes with other proteins, but also form hierarchical transcriptional regulation with other transcription factors. In *Artemisia annua*, the HD-Zip family proteins AaHD1 and AaHD8 directly activate the expression of MYB transcription factor *AaTAR2*, which can activate the expression of artemisinin biosynthesis pathway enzyme genes *ADS, CYP71AV1, DBR2* and *ALDH1*. Finally, AaTAR2 and HD-Zip synergistically promote the biosynthesis of artemisinin in *Artemisia annua* (Zhou et al., 2020).

## 6 Conclusion, current research questions and future prospects

The MYB gene family is a very important gene family in plants, and they are involved in plant organogenesis and growth, primary and secondary metabolite accumulation, and plant responses to biotic stresses. As one of the largest family of transcription factors in plants MYB transcription factors play a key role in plant response to abiotic stresses. Currently, scientists mainly focus on the response of MYB transcription factors to common abiotic stresses such as drought, cold, salt and heavy metals, so we summarized the genes in recent years that can be involved in the response to abiotic stresses by MYB transcription factors (**Table 3**). We also summarized the genes in which MYB transcription factors can affect plant growth and development and play a regulatory role in plant secondary metabolic processes (**Table 4**). In this review, we demonstrate that MYB transcription factors play a key role in response to abiotic stresses and that MYB transcription factors can also influence plant tolerance to abiotic stresses through hormonal pathways. As well, MYB transcription factors play important roles in plant secondary metabolic pathways (e.g. glucosides, flavonoids, terpenoids, lignans, and astragaloids).

**Table 3:** Abiotic stress responsive MYB transcription factors in plants.

Abiotic stress type	MYB transcription factors	Species	Target genes and sites	Reference
High temperature	AtMYBS1	*Arabidopsis thaliana* L.	*MAX1*	Li et al., 2023b
High temperature, salt, drought	AtMYB12	*Arabidopsis thaliana* L.	*ZEP, NCED, ABA2, AAO, P5CS*, *P5CR, LEA, SOD, CAT, POD* and Flavonoid biosynthesis genes	Wang et al., 2016
Salt	AtMYB25	*Arabidopsis thaliana* L.	*DREB2C, RD29a, SLAH1, JAZ10*	Beathard et al., 2021
Salt, drought	AtMYB37	*Arabidopsis thaliana* L.	*ABF2/3, COR15A, RD29a, RD22*, *PSII/I*	Li et al., 2022c
High temperature	AtMYB74	*Arabidopsis thaliana* L.	*ERF53, NIG1, HSFA6a, MYB47*, *MYB90, MYB102*	Ortiz-García et al., 2022
Drought	AtMYB94/96	*Arabidopsis thaliana* L.	*KCS1/2/6, KCR1, CER1/3, WSD1*	Lee et al., 2016
Salt, drought, cold	OsMYB2	Rice (Oryza sativa)	*OsLEA3, OsRab16A, OsDREB2A*	Yang et al., 2012
Salt, drought	OsMYB6	Rice (Oryza sativa)	*OsLEA3, OsDREB2A, OsDREB1A, OsP5CS*, *SNAC1, OsCATA*	Tang et al., 2019
Drought	OsMYB26	Rice (Oryza sativa)	*OsLEA3*	Chen et al., 2021c
Drought	OsMYB48-1	Rice (Oryza sativa)	*OsNCED4, OsNCED5*	Xiong et al., 2014
Drought	OsMYBR57	Rice (Oryza sativa)	*OsLEA3, Rab21*	Yang et al., 2022b
Drought	OsMYB60	Rice (Oryza sativa)	*OsCER1*	Jian et al., 2022
Salt	OsMYB91	Rice (Oryza sativa)	*SLR1*	Zhu N. et al., 2015
Cold	OsMYBS3	Rice (Oryza sativa)	*DREB1*	Su et al., 2010
Cold	OsMYB3R-2	Rice (Oryza sativa)	*DREB2A, COR15a, RCI2A*	Dai et al., 2007
Cold	OsMYB30	Rice (Oryza sativa)	*OsAGPL3, OsSSIIIb, OsSSIIb, OsSSIIc*	Lv et al., 2017
High temperature	OsMYB55	Rice (Oryza sativa)	*OsGS1, GAT1, GAD3*	El-Kereamy et al., 2012 Casaretto et al., 2016
Heavy metal stress	OsMYB30	Rice (Oryza sativa)	*Os4CL5*	Gao et al., 2023
Heavy metal stress	*OsARM1*	Rice (Oryza sativa)	*OsLsi1, OsLsi2, OsLsi6*	Wang et al., 2017a
Salt, drought	VhMYB2	*V. labrusca×V.* *riparia*	*SOS1/2/3, NHX1, SnRK2.6*, *NCED3, P5CS1, CAT1*	Ren et al., 2023
Salt, drought, cold	VaMYB14	*Vitis amurensis*	ABA signaling genes, CORs, LTPs, CAT, POD	Fang et al., 2024
High temperature	SlMYB41	*Solanum* *lycopersicum*	SlHSP90.3	Wang et al., 2023a
Drought	PsFLP	*Pisum sativum*	*CYCA2;3, CDKA;1, AAO3, NCED3*, *SnRK2.3*	Ning et al., 2023
Cold	BcMYB111	*Brassica campestris*	*F3H, FLS1*	Chen et al., 2023b
Drought	GhMYB36	*Gossypium hirsutum*	*PR1*	Liu et al., 2022
Salt	IbMYB308	*Ipomoea batatas*	*SOD, POD, APX, P5CS*	Wang et al., 2022a

**Table 4:** Functions of MYB transcription factors in the regulation of secondary metabolism.

Secondary metabolism	MYB transcription factors	Species	Reference
Promoting lignin biosynthesis	AtMYB20,42,43	*Arabidopsis thaliana*	Geng et al., 2020
Involvement in synthesis of flavonoids	GmMYB12	*Glycine max*	Wang et al., 2019a
Regulation of isoflavone biosynthesis	GmMYB176	*Glycine max*	Anguraj Vadivel et al., 2019
Positive regulation of flavonol biosynthesis	MdMYB22	*Malus domestica*	Wang et al., 2017b
Participation in lignin biosynthesis	ZmMYB167	*Zea mays*	Bhatia et al., 2019
Inhibition of anthocyanin synthesis	VvMYBC2L2	*Vitis vinifera*	Zhu et al., 2018
Regulation of biosynthesis of tomato cuticle wax	SlMYB31	*Solanum lycopersicum*	Xiong et al., 2020
Positive regulation of anthocyanin biosynthesis	SmMYB75	*Solanum melongena*	Shi et al., 2021
Negative regulation of anthocyanin synthesis	CmMYB#7/6	*Chrysanthemum morifolium*	Xiang et al., 2019
Inhibition of synthesis of lignin and flavonoids	CmMYB8	*Chrysanthemum morifolium*	Zhu et al., 2020
Positive regulation of anthocyanin biosynthesis	MrMYB1	*Myrica rubra*	Niu et al., 2010
Regulation of wound-induced anthocyanin accumulation	PdMYB118	*P. deltoides*	Wang et al., 2020a
Positive regulation of flavonol biosynthesis	PbMYB12b	*Populus canadensis*	Zhai et al., 2019
Positive regulation of anthocyanin biosynthesis	PyMYB10/114	*Pyrus pyrifolia*	Wang et al., 2020b
Inhibition of synthesis of lignin and polyphenol	MusaMYB31	*Musa nana*	Tak et al., 2017
Regulation of capsaicin biosynthesis	CaMYB31	*Capsicum annuum* var. *grossum*	Han et al., 2019
Regulation of citrus juice sac lignification	CsMYB330/308	*Celtis sinensis*	Jia et al., 2018
Participation in tanshinone and salvianolic acid metabolism	SmMYB98	*Salvia miltiorrhiza*	Hao et al., 2020
Negative regulation of anthocyanin biosynthesis	PtrMYB57	*Populus*	Wan et al., 2017
Participation in lignin biosynthesis	PtoMYB216	*Paulownia tomentosa*	Tian et al., 2013
Participation in seed oil synthesis	JcMYB1	*Jatropha carcas*	Khan et al., 2019
Inhibition of lignin deposition in xylem cell wall	EgMYB1	*Eucalyptus*	Soler et al., 2017
Overexpression enhances the synthesis and accumulation of anthocyanins	FtMYB1, FtMYB2	*Fagopyrum tataricum*	Bai et al., 2014
Inhibition of biosynthesis of rutin	FtMYB13, 14, 15, 16	*Fagopyrum tataricum*	Zhang et al., 2018
Participate in the synthesis of anthocyanidins in leaves	LhsorMYB12	*Lilium brownii* var. *viridulum*	Yamagishi et al., 2014
Inhibit the accumulation of phenolic acids	SmMYB39	*Salvia miltiorrhiza* *bunge*	Zhang et al., 2013
Participate in the biosynthesis of flavonoids under adverse conditions	GbMYB5, 26, 31	*Ginkgo biloba*	Liu et al., 2017
Negative regulation of the synthesis of lignin and flavonoids	CmMYB1	*Dendranthema morifolium*	Zhu et al., 2013
Regulates root structure, secondary wall biosynthesis and cellulose synthesis	OsMYB2P-1/OsMYB61	*Oryza sativa*	Dai et al., 2012 Chen et al., 2022b
Regulates cellulose and secondary wall synthesis affects leaf shape	OsMYB103L	*Oryza sativa*	Yang et al., 2014
Improve the biosynthesis and accumulation of lignin to improve rice resistance to brown planthopper	OsMYB30	*Oryza sativa*	He et al., 2020 Li et al., 2020b
Positive regulation of anthocyanin synthesis	OsMYB3	*Oryza sativa*	Zheng et al., 2021
Positive regulation of anthocyanin synthesis	OsC1	*Oryza sativa*	Upadhyaya et al., 2021
Activates genes encoding enzymes in the lithoxalate and cinnamate pathways, causing accumulation of ferulic acid	OsMYB110	*Oryza sativa*	Kishi-Kaboshi et al., 2018

Nowadays, many scientists have discovered various functions of MYB transcription factors. However, there are several shortcomings in studying the MYB family transcription factors in plants. Structure and classification of MYB: the family classification based on the number of MYB transcription factor domains is very rough. It lacks a brief description of its function. In the future, functional classification should be based on the functional differences of different family members, combined with structural and functional classification. This will help to better understand known MYB transcription factor genes and predict unknown MYB genes, thereby discovering more functions of MYB transcription factor genes.

MYB in hormone response to abiotic stress: scientists only focus on the changes in important plant hormones caused by MYB family transcription in plant related abiotic stress, but lack in-depth research on the regulatory mechanisms of MYB transcription factors in multiple hormone signaling pathways, and focus on studying their complex interaction mechanisms. In the future, scientists can pay more attention to how MYB transcription factors play a role at the intersection of ABA dependent and non dependent pathways in plant hormones to improve plant tolerance under abiotic stress.

The mode of action of MYB: Many scientists focus on the role of MYB TF in regulating single abiotic or biological stresses. This will cause multiple functional deletions of some MYB genes, leading to a lack of comprehensive understanding of the specific mechanism of action of MYB transcription factors in plants. Therefore, I believe that the TurboID technology can be used to screen multiple functional proteins that interact with MYB family transcription factors. With the continuous improvement of contemporary sequencing methods, ChIP-seq technology can be used to search for downstream target genes of transcription factors. Through the comprehensive application of these multiple technologies, we can better understand the mechanism of action of the multiple functions of MYB transcription factors.

MYB associated with abiotic stresses: Researchers currently only focus on the function of MYB transcription factor genes under abiotic stress, lacking research that combines MYB transcription factors with practical applications. Because our current research on the biological functions of genes is only at a basic level, our future research direction can combine the functional validation of MYB transcription factor genes with the practical application of stress resistant breeding to improve plant resistance and ultimately achieve high yields and stable yields in the future.

MYB regulate plant secondary metabolism: Current research on the regulation of plant secondary metabolites by MYB transcription factors is only linked to a single factor and abiotic stress, but there is little research exploring how light intensity affects the expression of MYB transcription factors, which in turn affects plant tolerance to abiotic stress and thus affects the level of plant regulation of secondary metabolites. If other environmental factors are introduced, we will be able to better understand how MYB transcription factors affect secondary metabolites and regulate abiotic stress. Due to varying yields of flavonols synthesized under light conditions. Therefore, in the future, we can focus on how different light intensities can have different effects on the expression of MYB transcription factors, thereby affecting the synthesis of flavonols and regulating abiotic stress.

So far, scientists have been conducting research on MYB transcription factors for over 30 years and have isolated and identified a large number of plant MYB TF families. However, most studies only focus on its structure, localization, gene expression regulation, and gene expression under stress, and do not delve particularly deeply into the function of MYB transcription factors. In the future, advanced biotechnological methods should be adopted to deeply study the mechanism by which MYB transcription factors regulate the production of plant secondary metabolites, comprehensively enhancing the stress resistance and economic value potential of crops. This is of great significance to the development of the agricultural economy. In addition, most of the research on MYB transcription factors has focused on the fields of crops, fruits and vegetables, while there are relatively fewer studies in the field of medicinal plants. The slow development of traditional Chinese medicine is largely due to the ambiguity of its mechanism of action. In the future, having a specific and clear understanding of the mechanism of action of MYB transcription factors will be equivalent to having a basic understanding of the micro-composition of traditional Chinese medicine. This will play an important role in further research on MYB transcription factors in medicinal plants, making it easier to study the structure, function and metabolic pathways of MYB transcription factors.

In summary, the growth and development of plants, the regulation of responses to abiotic stress and the secondary metabolic synthesis pathways of plants are all regulated by MYB transcription factors. Traditional breeding methods for improving plant traits are very time-consuming and can no longer meet the needs of modern plant breeding. In the future, controlling the expression of MYB family transcription factor genes through molecular breeding to improve plant traits, enhance the tolerance of plants to abiotic stress, and influence the secondary metabolism of plants through MYB transcription factors to cultivate more nutritious crop varieties will provide a theoretical basis for future biological breeding. Therefore, in-depth research on the molecular mechanisms of plant responses to abiotic stress, especially MYB transcription factors, involving all aspects of stress signal perception and transmission, transcriptional regulation, and expression of response genes, is aimed at ensuring the normal growth and development of plants under abiotic stress and enabling plants to synthesize secondary metabolites needed by humans, thereby ensuring high-quality food production. Ultimately, it will lay a solid foundation for future global food security and improving the quality of human life.
