# Supplementary material for: Structure, evolution, and roles of MYB transcription factors proteins in secondary metabolite biosynthetic pathways and abiotic stresses responses in plants: a comprehensive review
Source: Front Plant Sci. 2025 Jul 31;16:1626844. doi: 10.3389/fpls.2025.1626844 (PMC12350382; doi:10.3389/fpls.2025.1626844)

**SUPPORTING INFORMATION**

**Figure S1.** Phylogenetic tree MYBs family transcription factor proteins in *Arabidopsis thaliana*, *Oryza sativa*, *Glycine max, Panicum virgatum, Phragmites australis,* *Sorghum bicolor* and so on . Phylogenetic tree of MYB transcription factors proteins in selected angiosperms. The MYB family gene sequences of plants were retrieved using the NCBI (https://www.ncbi.nlm.nih.gov/) and China National Rice Data Center (https://www.ricedata.cn/). The phylogenetic tree was constructed using MEGA version 11.0 with the bootstrap method based on full amino acid sequences. Numbers next to the branches show the percentage of replicate trees in which the associated taxa clustered together in the bootstrap test (1000 replicates).


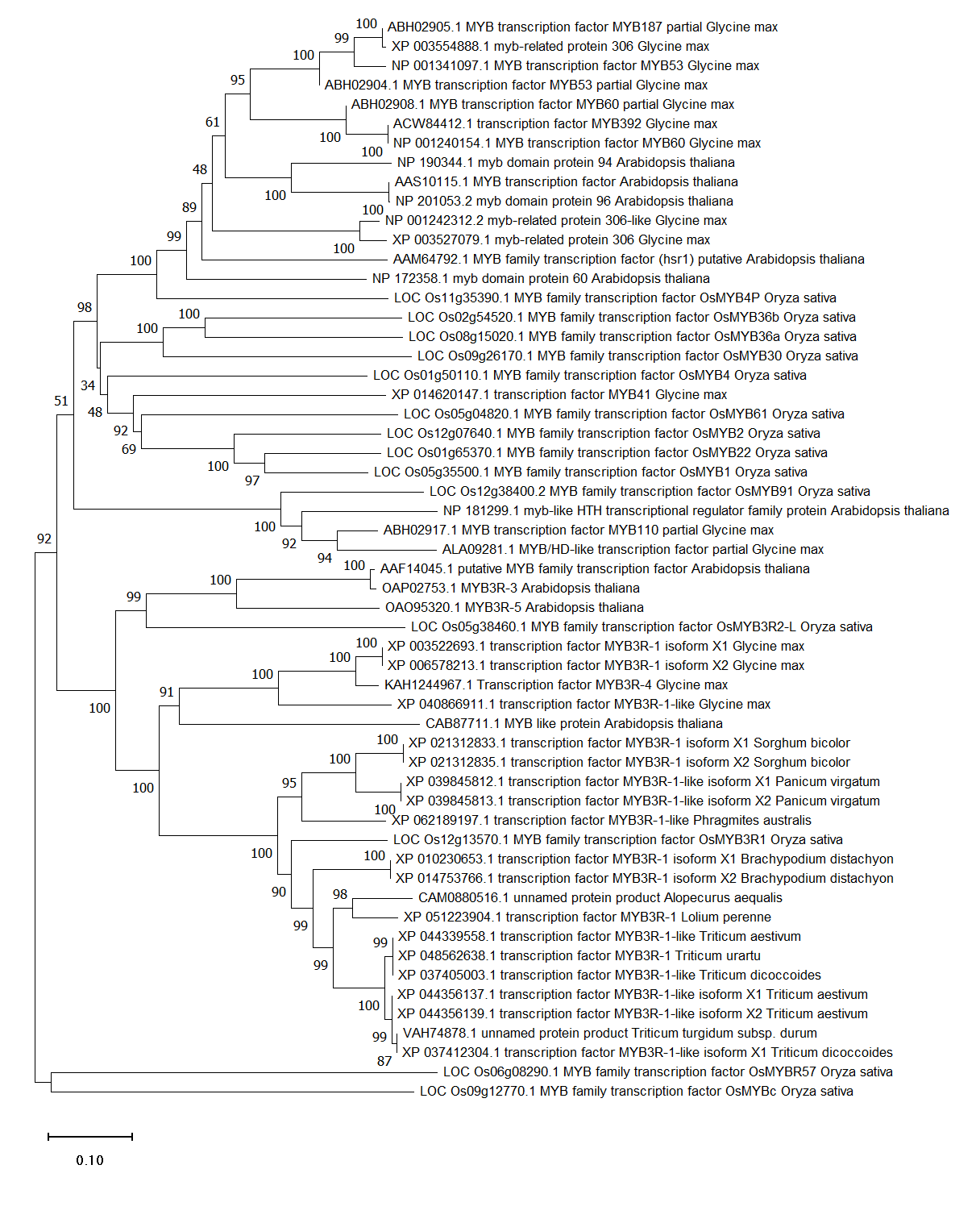

Supplement: Supplementary file 1 [file DataSheet1.docx]
